# Zhuyu pill relieves cholestasis in rats by modulating the expression of lncRNAs and circRNAs

**DOI:** 10.3389/fphar.2026.1744237

**Published:** 2026-09-29

**Authors:** Fenghua Zhang, Rui Cheng, Zhili Zheng, Zongying Xu, Jun Han, Han Yu

**Affiliations:** 1 School of Basic Medical Sciences, Chengdu University of Traditional Chinese Medicine, Chengdu, China; 2 Center for Post-Doctoral Studies, Guang’anmen Hospital, China Academy of Chinese Medical Science, Beijing, China; 3 Guang’anmen Hospital, China Academy of Chinese Medical Sciences, Beijing, China; 4 College of Basic Medical Sciences, Guangxi University of Chinese Medicine, Nanning, China

**Keywords:** cholestasis, inflammation, interventional mechanism, lipid metabolism, Zhuyu pill

## Abstract

**Background:**

Zhuyu pill (ZYP), a traditional Chinese medicine (TCM) formula composed of *Coptis chinensis* Franch and *Tetradium ruticarpum* (A. Jussieu) T. G. Hartley, exhibiting potential anti-cholestatic effects. However, its specific mechanism remains unclear. Long noncoding RNAs (lncRNAs) and circular RNAs (circRNAs) are critical gene expression regulators and promising direction for exploring the mechanism.

**Objective:**

This study aims to validate the anti-cholestatic effects of ZYP and explore its potential biological mechanisms through lncRNAs and circRNAs.

**Materials and methods:**

Rats with alpha-naphthyl isothiocyanate (ANIT)-induced cholestasis were treated with low-dose (0.6 g/kg) or high-dose (1.2 g/kg) ZYP. Efficacy was assessed by serum biochemistry and histopathology. Differentially expressed lncRNAs (DElncRNAs) and circRNAs (DEcircRNAs) in liver tissues were identified via RNA sequencing, followed by GO and KEGG enrichment analysis. MiRanda was used to predict DElncRNAs/DEcircRNAs-miRNA interactions and construct interaction networks. Subsequently, trend analysis was used to screen lncRNA/circRNA-miRNA pairs with expression patterns consistent with the competing endogenous RNA (ceRNA) regulatory mechanism.

**Results:**

Both high- and low-dose ZYP significantly improved biochemical and histopathological parameters, with the high-dose group showing superior effects. Enrichment analysis showed that DElncRNA-related functions were mainly concentrated in lipid metabolism pathways (“Bile secretion”, “Adipocytokine signaling pathway”, “Arachidonic acid metabolism”) and inflammation-related pathways (“NF-κB”, “TNF”, “MAPK”, and “Inflammatory bowel disease (IBD)”), suggesting their roles in alleviating inflammatory injury and lipid metabolism disorders. Among these, core lncRNAs altered by ANIT and reversed by ZYP included TCONS_00032614, TCONS_00018259, TCONS_00025702, TCONS_00012591, and TCONS_00003751, with qRT-PCR results largely consistent with sequencing data. Further lncRNA-miRNA binding prediction and expression trend analysis identified candidate pairs with potential competing ceRNA characteristics: TCONS_00003751/rno-miR-328b-3p, TCONS_00003751/rno-miR-328a-3p, and TCONS_00032614/rno-miR-149-5p. Similarly, DEcircRNA-associated pathways were enriched in inflammatory and immune responses (“Cell adhesion molecules (CAMs)”, “Antigen processing and presentation”, and “Cytochrome P450 metabolism of xenobiotics”). Candidate circRNAs included circRNA_1402 and circRNA_1452, and Candidate pairs with potential competing ceRNA characteristics included circRNA_1402/rno-miR-1306-5p and circRNA_1452/rno-miR-23a-5p.

**Conclusion:**

ZYP dose-dependently improved biochemical and histopathological parameters in cholestatic rats. This effect is closely related to the roles of lncRNAs and circRNAs in regulating lipid metabolism and inflammation-related pathways. Several candidate ceRNA pairs were preliminarily screened, yet further experimental validation is warranted.

## 1 Introduction

Cholestasis is an acute or chronic pathological phenomenon characterized by the accumulation of bile acids (BAs) in the liver and circulatory system, primarily resulting from impaired bile formation, secretion, and excretion (Pollock and Minuk, 2017; Shang et al., 2024). Its primary clinical manifestations typically include pruritus, fatigue, jaundice, and darkened urine (Lu, 2022). The pathological mechanisms of cholestasis include imbalances in bile acid metabolism, biliary obstruction, inflammation, and oxidative stress (Pessayre et al., 2010). Moreover, intestinal flora disorders can affect BAs via the enterohepatic axis (Xu et al., 2023). Cholestasis is classified as either intrahepatic or extrahepatic, depending on the region where it occurs (Luo and Lu, 2024). Cholestasis can lead to various harmful liver diseases, which are collectively named cholestatic liver disease (CLD). Among these, primary biliary cholangitis (PBC) and primary sclerosing cholangitis (PSC) are the major types (Pollock and Minuk, 2017). The morbidity of cholestasis continues to rise. It is estimated that there are about 19–402 cases per million people. (Lashgari et al., 2024). Moreover, CLD is a possible precursor to progressive hepatic fibrosis and subsequent cirrhosis. If left unmanaged, it significantly elevates the risk of developing hepatobiliary malignancies, particularly hepatocellular carcinoma (Contreras-Omaña et al., 2022; Yu et al., 2023). Currently, cholestasis has emerged as a significant public health challenge, adversely impacting both health and quality of life. However, therapeutic options for cholestasis and its associated CLD remain severely limited. Ursodeoxycholic acid (UDCA) is the only first-line therapeutic agent clinically recommended for PBC patients, yet there exists a substantial population of UDCA non-responders (Floreani and Mangini, 2018; Gallucci et al., 2024). Moreover, apart from liver transplantation, there are currently no effective treatment options available for PSC (Santiago et al., 2018). The clinical treatment of cholestasis urgently requires potentially effective drugs and new therapeutic regimens.

Traditional Chinese Medicine, a unique medical resource in China with a 3,000-year legacy, is characterized by its stable therapeutic efficacy, minimal adverse reactions, and multi-target synergistic effects. Its botanical drug formulations offer a promising avenue for uncovering actionable molecular targets and developing targeted therapies for cholestasis management. According to TCM theory, the primary pathological mechanism of cholestasis is internal accumulation of damp-heat in the liver and gallbladder system. This pathogenic pattern can lead to obstruction of the liver and gallbladder *qi*. *Qi* can maintain the normal function of the liver and gallbladder system, which is crucial for promoting the generation and excretion of bile (Liang et al., 2020). ZYP is a classic formula with the therapeutic principle of removing dampness-heat and dispersing stagnated liver and gallbladder *qi*, targeting the pathogenesis of cholestasis. ZYP was first published in the traditional Chinese medical text *Tai Ping Sheng Hui Fang* (Han, 2010). It consists of a 1:1 ratio of *Coptis chinensis* Franch. (Huanglian in Chinese) and *Tetradium ruticarpum* (A. Jussieu.) T. G. Hartley (Wuzhuyu in Chinese). The main metabolite of *Coptis chinensis* is berberine, and the main metabolites of *Tetradium ruticarpum* are dehydroevodiamine (DeHE), evodiamine (Evo), rutaecarpine (Rut), etc (Wang J. et al., 2019). Both the active metabolites of individual drugs and their combination have demonstrated the ability to regulate cholesterol and bile acid synthesis, alleviate hepatic inflammation, exert antioxidant and antitumor effects, and modulate intestinal flora and the intestinal immune response. (Yu et al., 2023; Wang J. et al., 2019; Jia et al., 2021). Previous studies have confirmed the therapeutic effects of ZYP on cholestasis and ulcerative colitis (Yu et al., 2022). The mechanisms have also been preliminarily interpreted in terms of fecal microbial homeostasis and hepatic mRNA and miRNA expression (Han et al., 2023; Yu et al., 2021). However, research elucidating the specific mechanisms by which ZYP treats cholestasis from the perspectives of lncRNAs and circRNAs remains unexplored.

LncRNAs are a class of non-coding RNA transcripts that are more than 200 nucleotides in length and cannot code for proteins. However, lncRNAs can regulate gene expression through multiple mechanisms, including acting as “molecular sponges” to sequester microRNAs (miRNAs), interacting with proteins, performing epigenetic modifications, etc (Arraiano, 2021; Yip et al., 2021). CircRNAs are a specialized class of lncRNA molecules with a circular structure formed by reverse splicing (Chen et al., 2021). Compared to linear RNAs, its longer half-life and stronger RNase R resistance make it more advantageous as a diagnostic biomarker and therapeutic target (Zhu et al., 2021). Evidence from emerging studies has demonstrated that aberrant expression of lncRNAs and circRNAs is significantly associated with various liver diseases, encompassing their onset, progression, diagnosis, and treatment. For cholestasis, Liu et al. demonstrated that lncRNA-H19 could regulate bile stasis and subsequent hepatic fibrosis (Liu et al., 2019). Multiple differential expressions of lncRNA and circRNA were found in intrahepatic cholestasis of pregnancy (Xiong et al., 2023). Chsa-circ-402458 was proved to regulate inflammatory and immunological responses of PBC through miRNA sponge action (Zheng et al., 2017). In conclusion, lncRNAs and circRNAs are potential targets that cannot be neglected when exploring the mechanism of ZYP in the treatment of cholestasis.

## 2 Materials and methods

### 2.1 Chemicals and materials

The two Chinese botanical drugs used in the production of ZYP, Huanglian and Wuzhuyu, were sourced from Beijing Tongrentang Co., Ltd. (Beijing, China). UDCA, sodium pentobarbital, 0.5% CMC-Na solution, and alpha-naphthyl isothiocyanate were obtained from Sigma-Aldrich Co. (St. Louis, MO, USA). Olive oil was selected from Shanghai Yi En Chemical Technology Co., Ltd. (Shanghai, China). For this experiment, all chemicals used were of high-performance liquid chromatography (HPLC) analytical grade.

### 2.2 The preparation and identification of ZYP

The characteristics of the two botanical drugs are presented in Table 1. ZYP preparation was produced following the Science of Prescription guidelines outlined in the Ministry of Education’s “13th Five Year Plan” for Higher Education national planning materials. Dried botanical drugs, *Coptis chinensis* Franch. and *Tetradium ruticarpum* (A. Jussieu) T. G. Hartley, were mixed at a 1:1 ratio (w/w), immersed in 20-fold volumes of purified water (v/w) for 30 min, then heated to boiling and maintained for 30 min. The decoction was filtered and collected. The drug residues were boiled again using the method described above; the resulting liquid was collected, combined with the initial liquid, and the mixture was concentrated to 120 mL. After filtration, the solution was concentrated under reduced pressure to a suspension with a final concentration of 0.1 g/mL and stored at −20 °C.

**TABLE 1:** Composition of Zhuyu pill.

Chinese name	Botanical name^a^	Genus family	Batch number	Medicinal parts	Origin	Weight(g)
Huanglian	*Coptis chinensis* Franch	Ranunculaceae	220,701	Dried root	Chongqing, China	6
Wuzhuyu	*Tetradium ruticarpum* (A. Jussieu.) T. G. Hartley	Rutaceae	220416008	Dried mature seed	Guizhou, China	6

^a^ The plant name originates from the standardized nomenclature on the http://www.theplantlist.org.

The major active metabolites of ZYP were quantified by HPLC. Four primary metabolites were identified: berberine (36.8 mg/g), phellodendrine (14.9 mg/g), glutaralbine (0.78 mg/g), and rutecarpine (0.33 mg/g). Moreover, the fingerprints of ZYP showed a consistent trend, which ensured the homogeneity and stability of its intrinsic quality. The detailed preparation procedure and HPLC analysis process for ZYP are specifically documented in Supplementary Note S1, and its fingerprint is shown in Supplementary Figure S1. The complete ConPhyMP checklists for this study are available in Supplementary Data Sheet 1 and Supplementary Data Sheet 2 of the supplementary materials.

### 2.3 Ethics approval

Experiments were conducted in strict accordance with internationally recognized guidelines for the care and use of laboratory animals. And we have been approved by the Animal Ethics Committee of Chengdu University of Traditional Chinese Medicine (Ethics Approval No. 2019-15).

### 2.4 Animals and treatments

After a 4-day acclimatization period, thirty healthy male rats (160-180 g) were randomly divided into five groups of six (n = 6). According to the different treatments, the groups were designated as follows: the control group (Control), the model group (Model), the ZYP low-dose group (ZYP-L), the ZYP high-dose group (ZYP-H), and the ursodeoxycholic acid group (UDCA). All animals were procured from Beijing HFK Bioscience Co., Ltd (Certification No.: SCXK-JING, 2019-0008).

The dosing regimen for rats was determined using a body surface area (BSA) scaling method, which complies with the U.S. Food and Drug Administration (FDA) guidelines for human-to-animal dose extrapolation (U.S. Food and Drug Administration, 2005). Based on the body surface area ratio of rats, the standardized dose of the experimental drug should be 1.2 g/kg, which translates to 12 g/60 kg in humans. During the first phase (Days 5-10), rats in the ZYP-L and ZYP-H groups were administered ZYP via gavage at doses of 0.6 g/kg and 1.2 g/kg, respectively. Meanwhile, the UDCA group received UDCA (60 mg/kg/day) suspended in 0.5% CMC-Na solution. (Ding et al., 2008; Yang et al., 2018; Han et al., 2023). The model and control groups were given an equivalent volume of purified water. In the second phase (Day 11), except for the control group, which received 50 mg/kg of pure olive oil, all experimental groups were administered 50 mg/kg of alpha-naphthyl isothiocyanate (ANIT) solution dissolved in olive oil by gavage to induce cholestasis. In the third phase (Days 12-14), ZYP at 0.6 g/kg and 1.2 g/kg, and UDCA at 60 mg/kg were administered to rats. were used to treat rats in the ZYP-L, ZYP-H, and UDCA groups, respectively. Equal amounts of purified water were given to rats in the model and control groups. At the end stage (Day 15), all groups of rats were anesthetized and sacrificed with 150 mg/kg sodium pentobarbital. Blood and liver tissues were then collected from each group for further analysis. The specific experimental steps are shown in Figure 1.

**FIGURE 1:**
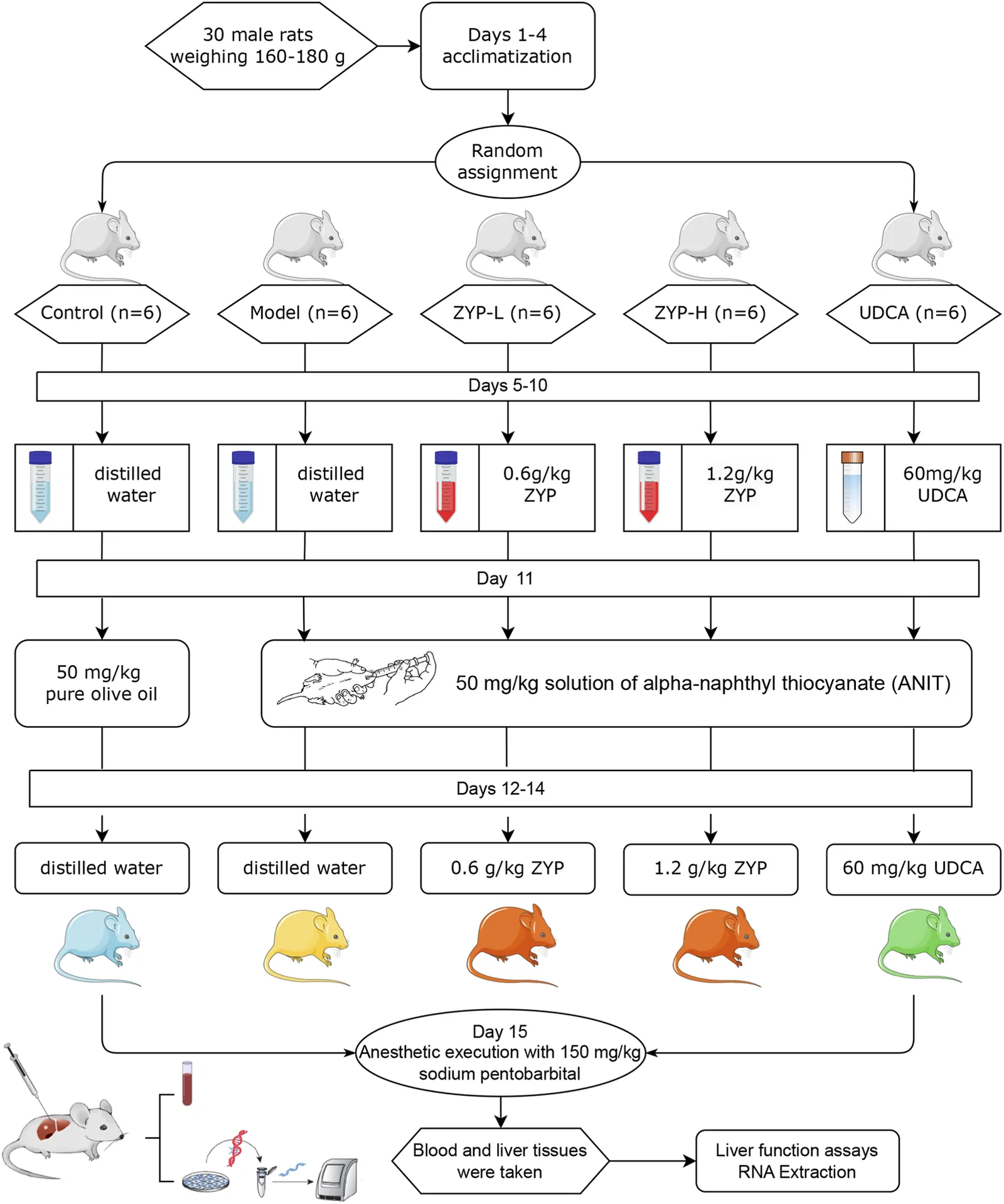
Experimental procedure.

### 2.5 Liver function and histopathological evaluation

Following a 12-h fasting period, blood and liver tissue samples were collected from rats under sodium pentobarbital-induced anesthesia. Blood samples were centrifuged at 3500 *g* for 15 min at 4 °C to obtain serum samples. Serum levels of alanine aminotransferase (ALT), aspartate aminotransferase (AST), alkaline phosphatase (ALP), γ-glutamyl transferase (γ-GT), direct bilirubin (DBIL), total bilirubin (TBIL), total bile acids (TBA), triglycerides (TG), and total cholesterol (TC) were quantitatively analyzed using a fully automated biochemical analyzer (BS-240VET). Meanwhile, hematoxylin and eosin (H&E) staining was performed on liver tissue specimens after paraffin section processing. (specific procedure: 4% paraformaldehyde perfusion to achieve fixation, tap water rinsing, dehydration, embedding, sectioning, H&E staining). The stained sections were observed under a microscope, and histopathological changes were further analyzed.

### 2.6 QRT-PCR validation

Total RNA was extracted from liver tissue samples and processed by removing ribosomal RNA (rRNA) with a ribo-zero kit, followed by fragmentation into short segments. Using random six-base primers, the RNA was reverse-transcribed into single-stranded cDNA, which was then converted into double-stranded cDNA with dUTP instead of dTTP. Next, different adapters were ligated, and the DNA strands containing dUTP were digested with uracil-N-glycosylase (UNG), thereby retaining the DNA strands bearing different adapters. The cDNA strands were purified and then sequentially subjected to end repair, addition of A-tail, ligation of sequencing adapters, fragment screening, and PCR amplification. Finally, the library was quality-checked using the Agilent 2100 Bioanalyzer (Agilent Technologies, Santa Clara, CA, USA) before being subjected to sequencing on the Illumina platform. The list of specific primers employed is presented in Table 2.

**TABLE 2:** QRT-PCR primers used in this study.

Gene Symbol	Forward primer (5->3)	Reverse primer (5->3)	Product length (bp)	Tm (°C)
TCONS_00032614	GTG​ATA​AAC​CAG​GTG​ATC​GG	AAGGCGAAGCTACAAACT	102	60
TCONS_00018259	GGC​ATA​TAA​CCA​TAC​CAG​ATC​G	GCA​TGA​TGT​CAA​CCA​AGG​TAG	102	60
TCONS_00012591	TAA​ACA​TGC​ACC​ACT​GGA​TGT​A	CCT​CAA​TCC​ACA​GGA​AAT​CCT​A	162	60
TCONS_00025702	TCT​CAG​AGC​AAG​ACA​CAG​C	TCA​AGA​TTA​GGC​AAG​GCC​A	80	60
TCONS_00003751	CAGCAAGGTCACATGCAG	GATGCCTTGCCTACAGTT	112	60

### 2.7 Data preprocessing and genome comparison, transcript splicing

First, rRNA sequences were removed using SortMeRNA (Kopylova et al., 2012). Subsequently, adapters, reads containing ambiguous bases (e.g., “N” nucleotides), low-quality reads, and reads shorter than 50 bp were filtered out using Trimmomatic (Bolger et al., 2014). The filtered results were then quality checked using fastp (Chen et al., 2018), and the final clean reads obtained were compared to the rat reference genome using Hisat2 (Kim et al., 2015). The comparison quality was assessed by RSeQC (Wang et al., 2012), and the complete transcripts were assembled and spliced using StringTie (Pertea et al., 2015).

### 2.8 Prediction, quantification, and differential analysis of lncRNAs and circRNAs

The results of transcript assembly were screened to obtain the candidate lncRNA collection, the specific procedure of which is described in Supplementary Note S2. Clean reads were aligned to transcript templates using Bowtie2 (Langmead and Salzberg, 2012) (http://bowtie-bio.sourceforge.net/bowtie2/manual.shtml), and transcript abundance was quantified using eXpress (Roberts and Pachter, 2012) to obtain FPKM values and read counts. For circRNA prediction, clean reads were first aligned to the reference genome using BWA (Li and Durbin, 2009). Based on the resulting SAM file, circRNAs were predicted by CIRI (Gao et al., 2015) (https://github.com/bioxfu/circRNAFinder). The detailed flow is provided in Supplementary Note S3.

Differential expression analysis was performed using DESeq (Anders, 2011). Following normalization by estimateSizeFactors and significance testing by nbinomTest, the false discovery rate (FDR) was controlled by the Benjamini-Hochberg method. Differentially expressed lncRNAs and circRNAs were identified using the following criteria: adjusted *p*-value (FDR) < 0.05 and |log_2_foldchange| > 1 (see Supplementary Note S4 for details).

### 2.9 GO and KEGG functional analysis

GO functional enrichment analysis and KEGG pathway enrichment analysis were conducted using clusterProfiler (Yu et al., 2012) and the Oebiotech Cloud Platform (https://cloud.oebiotech.cn/task/). Enrichment analysis was performed using the hypergeometric distribution test. Enrichment significance was assessed by *p*-values, which were subsequently corrected using the Benjamini-Hochberg method to obtain false discovery rate (FDR).

### 2.10 Prediction of circRNA/lncRNA-miRNA interactions and network construction

CircRNAs and lncRNAs that bind to miRNAs were identified using miRanda (Enright et al., 2003), and their functions were elucidated based on functional annotations of miRNA target genes. Predicted lncRNA-miRNA binding interaction network were subsequently constructed. Network constructions were performed using the Oebiotech Cloud Platform (https://cloud.oebiotech.cn/task/).

### 2.11 Preprocessing of expression profiles

Core lncRNAs and circRNAs in key pathways were paired with their predicted high-binding-affinity miRNAs to form potential ceRNA pairings. The corresponding circRNA/lncRNA and miRNA expression matrices were then extracted from RNA sequencing data of the Control, Model, and ZYP_H groups (3 biological replicates per group). The arithmetic mean of each group was calculated as the statistical metric for inter-group expression comparison. Molecules with zero expression across all three groups were excluded as non-expressed transcripts, and invalid data pairs were filtered out before subsequent trend analysis.ceRNA.

### 2.12 Regulatory trend determination and visualization

According to ceRNA hypothesis, circRNA/lncRNA exerts a negative regulatory effect on miRNA expression levels through sponge adsorption; thus, the expression levels of the two should exhibit dynamic negative correlation. This study screened ceRNA pairing candidates through two critical nodes, “modeling” (Model vs. Control) and “treatment” (ZYP_H vs. Model): circRNA/lncRNA and miRNA were required to exhibit completely opposite expression change directions at both nodes. Pairs that simultaneously satisfy the negative direction of change are considered core candidate pairs for subsequent analysis.

Paired molecular inter-group expression trend line plots were generated using GraphPad Prism version 8. The X-axis represents the grouping (Control/Model/ZYP_H), and the Y-axis represents the mean expression level, with error bars denoted as mean ± standard deviation (Mean ± SD). For pairs in which the expression abundances of circRNA/lncRNA and miRNA differ markedly, a dual-Y-axis (left and right axes independent) plot would be used to display the expression changes of the two types of molecules separately.

### 2.13 Screening of core candidate ceRNA pairings

The intersection of “high-binding-affinity predicted pairings” and “ceRNA expression trend-conforming pairings” was extracted as the core candidate ceRNA regulatory pairings for this study.

### 2.14 Statistical analysis

Comparison of serum biochemical indices was performed by GraphPad Prism version 8. The results of the experimental data were expressed as mean ± standard deviation.Differences among groups were assessed using one-way analysis of variance (ANOVA), combined with Tukey’s *post hoc* test for pairwise comparisons. The *p-*values <0.05 means the difference is statistically significant.

## 3 Results

### 3.1 Effect of ZYP on hepatic function and pathology

Biochemical analysis showed marked elevations in hepatobiliary injury markers (ALT, AST, ALP, γ-GT) and metabolic dysregulation indicators (DBIL, TBIL, TBA, TC, TG) in cholestatic rats compared with control animals (Figures 2A–I). Treatment with either ZYP-L or ZYP-H significantly attenuated these pathological increases, with ZYP-H exhibiting superior efficacy over ZYP-L in normalizing serum parameters. It reflected a dose-responsive therapeutic benefit. Moreover, ZYP treatment significantly reduced elevated lipid markers (TC, TG) in the model group, indicating effective modulation of cholestasis-associated lipid metabolism.

**FIGURE 2:**
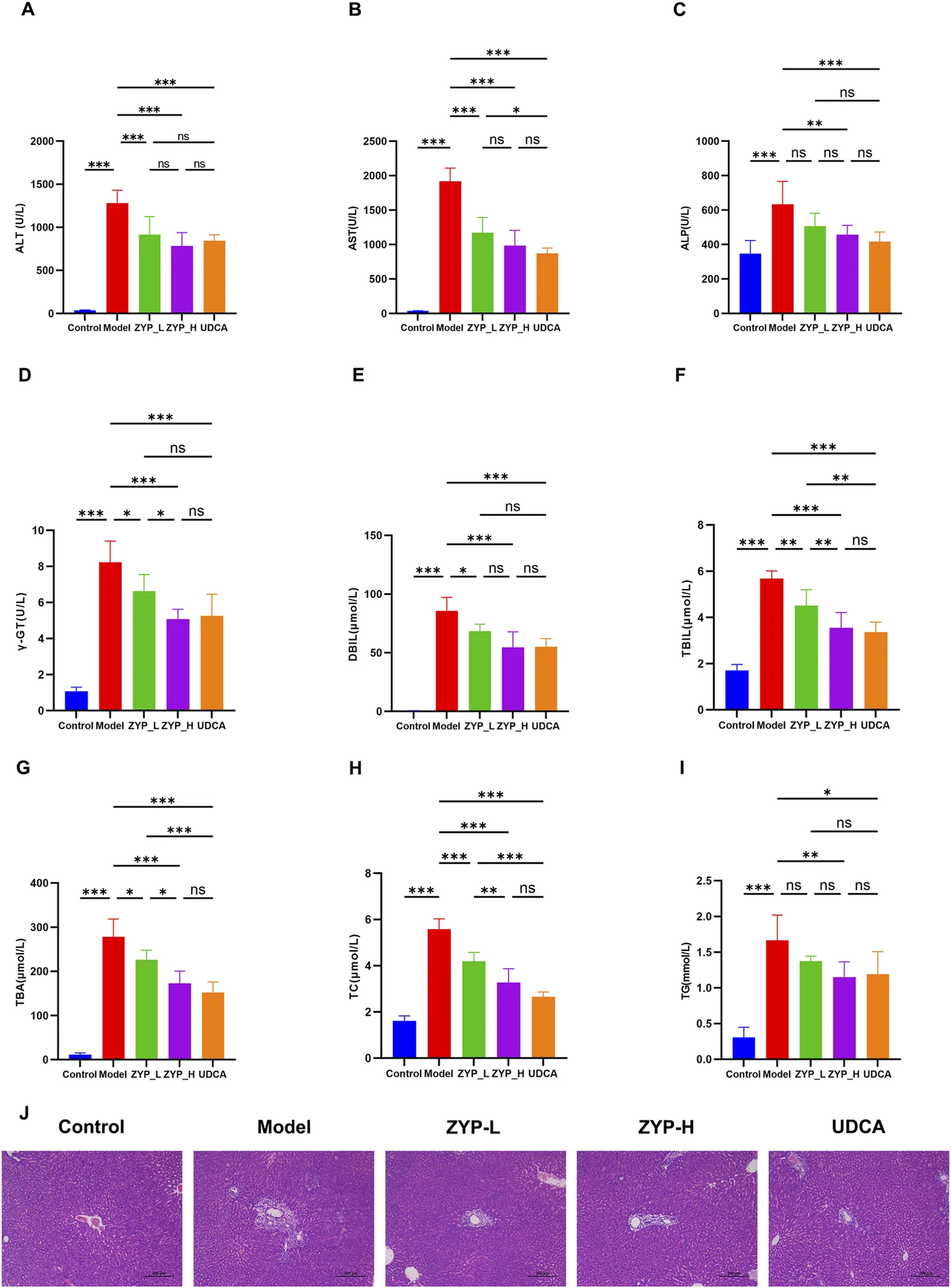
Serum biochemical indices and histopathological changes in the liver tissues of each group. **(A)** ALT, **(B)** AST, **(C)** ALP, **(D)** γ-GT, **(E)** DBIL, **(F)** TBIL, **(G)** TBA, **(H)** TC, **(I)** TG. **(J)** Representative histopathological images of liver sections (hematoxylin and eosin staining, ×200 magnification), ***, p < 0.001, **, p < 0.01, *, p < 0.05, ns, not significant.

Complementing these biochemical findings, histopathological analysis (Figure 2J) revealed normal hepatic architecture in controls, characterized by orderly arranged hepatocytes, well-defined cellular boundaries, abundant cytoplasm, uniform staining, regularly spherical nuclei, and preserved venous endothelial integrity. The model group exhibited focal necrosis with inflammatory infiltrates. Low-dose ZYP was already capable of significantly alleviating inflammation and restoring tissue architecture; high-dose ZYP further enhanced this therapeutic efficacy, bringing histological presentation closer to the normal state. This dose–response relationship indicates that ZYP exerts genuine therapeutic efficacy against liver injury—it is not a marginally effective agent that only works at high doses. Its effect is dose-dependent, and rational dose escalation holds promise for achieving superior treatment outcomes. ZYP-induced histological recovery was comparable to UDCA treatment. The concordance between serum biomarker normalization and structural preservation confirmed the ability of ZYP to alleviate cholestasis-induced liver injury and restore metabolic regulation.

### 3.2 Screening and quantitative statistics of differentially expressed lncRNAs (DElncRNAs)

As shown in Figure 3A, a total of 3,332 lncRNAs were predicted as potential targets by the four prediction tools. The PCA plot (Figure 3B) revealed a significant distinction between the model group and the control group, suggesting that cholestasis induced alterations in the global lncRNA expression profile. Both ZYP-H and ZYP-L groups were positioned between the Control and Model groups along the PC1 axis (37.65% variance). This pattern indicates that ZYP intervention conferred a therapeutic benefit in normalizing disease-related transcriptional profiles at both dosage levels. The ZYP-H group centroid was closer to the Control group than that of the ZYP-L group, suggesting a potential dose-effect trend (Figure 3B). DElncRNAs were determined based on |log_2_foldchange| > 1 and adjusted *p*-value <0.05. Compared with the control group, ANIT-induced cholestasis was associated with significant alterations in 852 lncRNAs (422 upregulated and 430 downregulated; Figure 3C). Relative to the model group, ZYP-L treatment resulted in 169 DElncRNAs (98 upregulated and 71 downregulated; Figure 3D), whereas ZYP-H treatment resulted in 146 DElncRNAs (75 upregulated and 71 downregulated; Figure 3E). Notably, ZYP administration markedly reduced the number of dysregulated lncRNAs in a dose-dependent manner.

**FIGURE 3:**
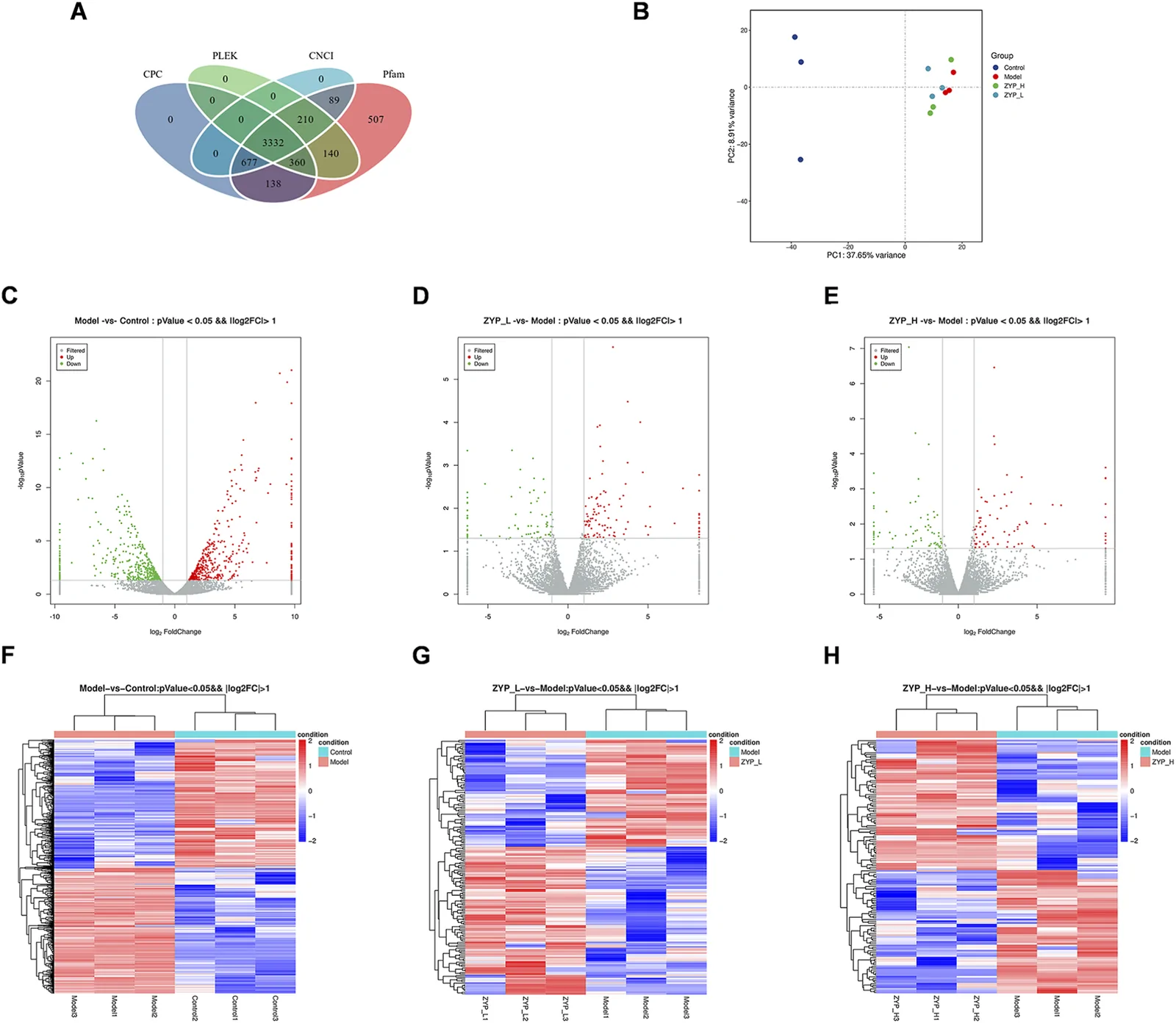
lncRNA Sequencing Analysis. **(A)** Venn diagram showing the results of non-coding transcripts obtained from different prediction tools. **(B)** Principal component analysis (PCA) plot reflecting the clustering status among samples and overall differences. **(C–E)** Volcano plots reflecting gene expression differences. |log_2_foldchange| > 1 and p < 0.05 were set as the criteria for significance, with non-significant genes shown in gray, significantly upregulated genes in red, and significantly downregulated genes in green. **(F–H)** Heatmap representing gene expression patterns and hierarchical clustering. The x-axis represents samples, and the y-axis represents genes. Expression levels are color-classified: white shows no intergroup difference, red shows high expression, and blue shows low expression. The dendrogram on the left illustrates clustering of genes.

The overall color distribution of the heatmaps (Figures 3F–H) indicates that ZYP treatment exerts bidirectional modulation of lncRNA expression in the cholestasis model: lncRNAs induced by ANIT were suppressed by ZYP, while those downregulated by ANIT were reactivated. Given the interventional advantage of the high-dose group (ZYP-H), we focused on the DElncRNAs regulated by ZYP-H. Specifically, the study prioritized lncRNAs that were upregulated in the model group but downregulated in the ZYP-H group, as well as those downregulated in the model group but upregulated in the ZYP-H group. These DElncRNAs more accurately represented the corrective effects of ZYP.

### 3.3 Enrichment analysis of DElncRNAs regulated by ZYP

To explore the biological functions of the DElncRNAs, functional enrichment analysis was conducted. As shown in Figure 4A, GO analysis revealed that the terms co-enriched by lncRNAs downregulated in the model group and upregulated in the ZYP-H group were significantly associated with immune and inflammatory regulation, glycolipid metabolism, stress response, and substance transport in the Biological Process (BP) category. In the Cellular Component (CC) category, enriched terms were primarily related to organelle membranes. For Molecular Function (MF), the terms were mainly involved in glycolipid metabolism and transporter activity. Consistent with the GO findings, KEGG pathway analysis (Figure 4C) highlighted enrichment of lncRNAs in glycolipid metabolism, inflammatory response, stress reaction, and bile acid metabolism. Among these, pathways closely associated with cholestasis included “Bile secretion”, “Adipocytokine signaling pathway”, “NF-κB signaling pathway”, and “Arachidonic acid metabolism.”

**FIGURE 4:**
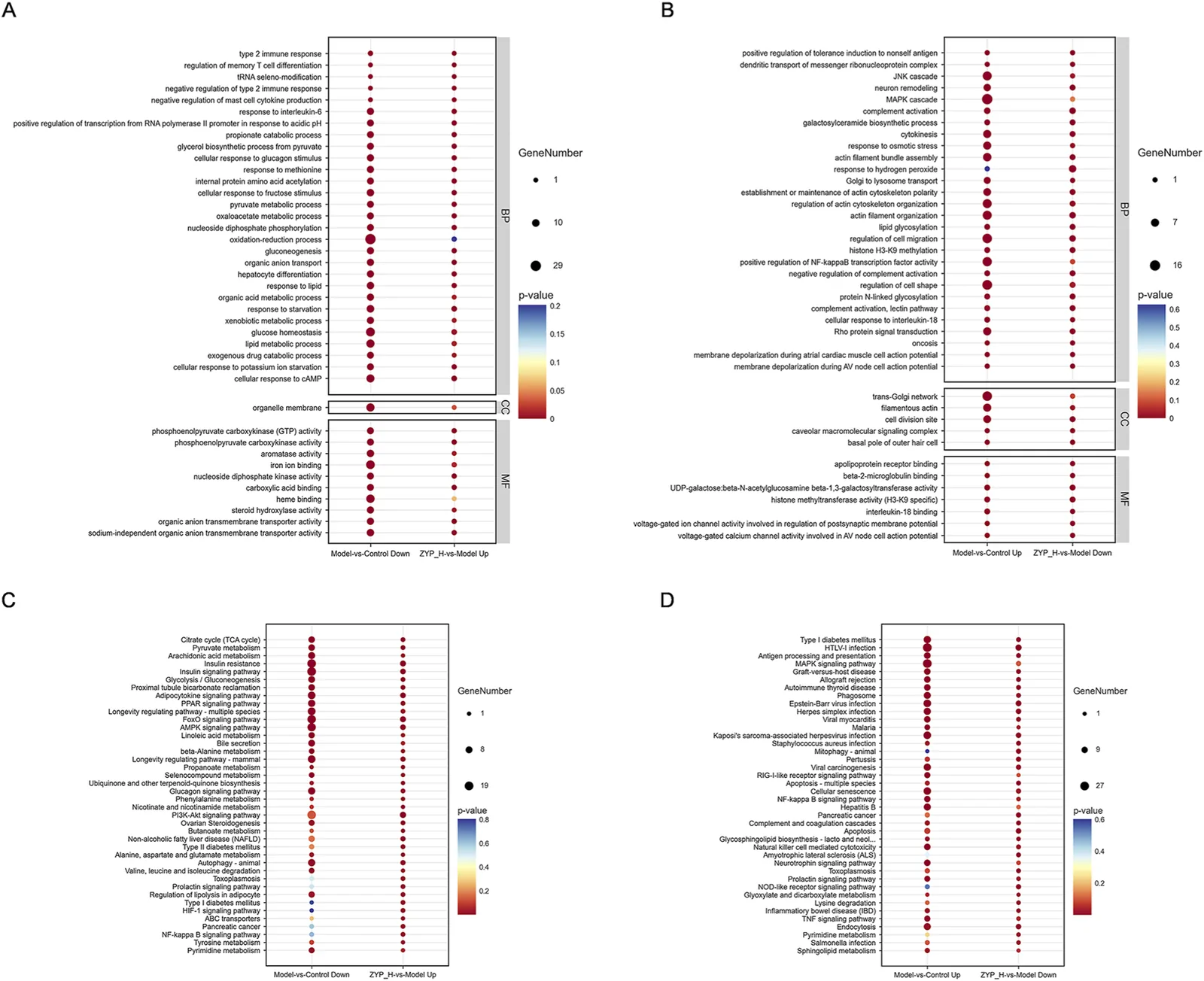
Functional enrichment analysis of DElncRNAs. GO Analysis: **(A)** Top 40 GO terms co-enriched by lncRNAs that were downregulated in the model group and upregulated after ZYP treatment. **(B)** Top 40 GO terms co-enriched by lncRNAs that were upregulated in the model group and downregulated after ZYP treatment. KEGG Analysis: **(C)** Top 40 signaling pathways co-enriched by lncRNAs that were downregulated in the model group and upregulated after ZYP treatment. **(D)** Top 40 signaling pathways co-enriched by lncRNAs that were upregulated in the model group and downregulated after ZYP treatment.

For GO terms co-enriched by lncRNAs that were upregulated in the model group and downregulated in the ZYP-H group (Figure 4B), enriched BP entries were associated with immune and inflammatory responses, glycolipid metabolism, stress response and cell death, cellular structure, and substance transport. CC entries were primarily related to substance transport, structural support, and signal transduction, while MF entries were linked to molecular binding and recognition, catalytic activity, and membrane transport. Similarly, as shown in Figure 4D, the signaling pathways were associated with immune and inflammatory responses, stress responses, and cell death. Among these, the KEGG pathways most closely related to cholestasis included “NF-κB signaling pathway”, “TNF signaling pathway”, “MAPK signaling pathway”, and “Inflammatory bowel disease (IBD)”.

In these pathways, “Bile secretion”, “Adipocytokine signaling pathway”, and “Arachidonic acid metabolism” all point to lipid metabolism processes. Previous studies have indicated that lipid metabolism disorders may underlie the pathogenesis of cholestasis (Wang B. L. et al., 2019). Consequently, lipid metabolism-related signaling pathways have emerged as potential therapeutic targets for treating cholestasis. In addition, the inflammatory and immune-related pathways, including “NF-κB signaling pathway”, “TNF signaling pathway”, “MAPK signaling pathway”, and “inflammatory bowel disease (IBD)”, accounted for a high proportion. Given that inflammatory cascades and immune responses triggered by toxic BAs are critical in the progression of cholestasis, these pathways are also considered key therapeutic targets. Interestingly, both upregulated and downregulated lncRNAs were enriched in the “Adipocytokine signaling pathway”, “NF-κB signaling pathway”, “TNF signaling pathway”, and “MAPK signaling pathway”, implying that these pathways may be modulated by lncRNAs in opposing directions (Figure 5). Overall, the biological processes and signaling pathways regulated by ZYP-H in the treatment of cholestasis are mainly concentrated in lipid metabolism, inflammation, and immune responses, all of which are closely associated with the pathological mechanisms of cholestasis. Thus, these seven signaling pathways have become the focus of our research.

**FIGURE 5:**
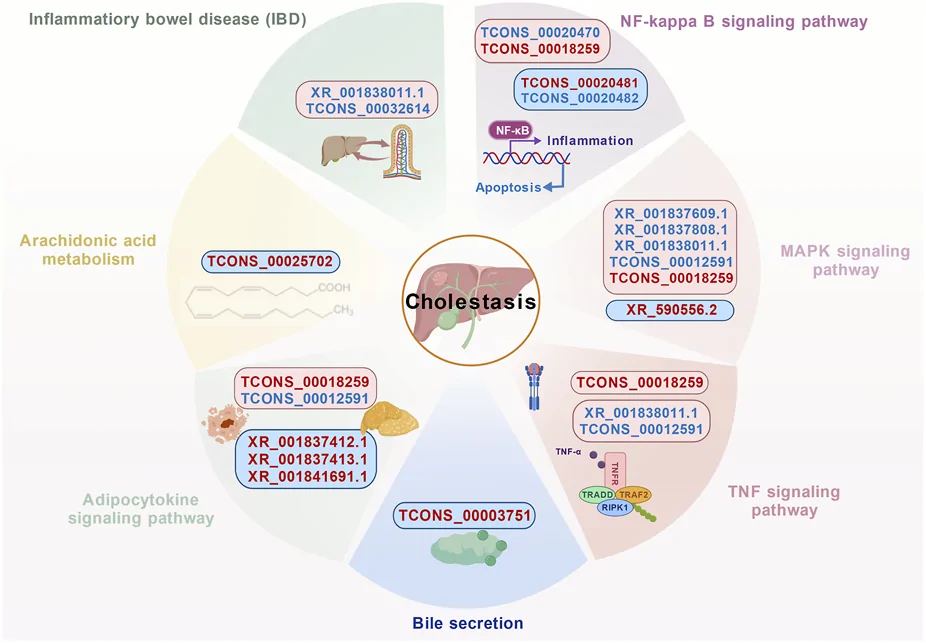
Mechanistic network of ZYP regulation in cholestasis. The mechanistic network encompasses seven major pathways: Bile secretion, Adipocytokine signaling pathway, Arachidonic acid metabolism, Inflammatory bowel disease (IBD), TNF signaling pathway, MAPK signaling pathway, and NF-κB signaling pathway. Compared with the control group, lncRNAs marked with blue boxes in the model group were significantly downregulated, while those in red boxes were upregulated. Compared with the model group, lncRNAs in blue font represent downregulation in the ZYP-H group, while those in red font indicate upregulation.

### 3.4 Regulatory mechanisms of ZYP in the treatment of cholestasis

To elucidate the mechanism and key therapeutic targets of ZYP in the treatment of cholestasis, this study integrated the lncRNA regulatory profiles of seven major signaling pathways identified through screening and further constructed a simplified mechanism diagram (Figure 5) using BioGDP.com (Jiang et al., 2024). The mechanistic diagram showed that, compared with the control group, the cholestasis model exhibited obvious dysregulation in relevant pathways, whereas ZYP-H exhibited comprehensive antagonistic regulatory effects on these pathological axes. Among the lncRNAs depicted in the mechanistic diagram, TCONS_00032614, TCONS_00025702, TCONS_00003751, TCONS_00018259, and TCONS_00012591 were identified as key candidate therapeutic targets.

### 3.5 QRT-PCR validation of key lncRNAs

As shown in Figure 6, the expression trends of all lncRNAs, except TCONS_00018259, aligned with the RNA-seq data, confirming the reliability of the sequencing results. These findings further substantiate that ZYP can reverse the aberrant gene expression induced by ANIT. TCONS_00018259 showed inconsistent expression trends between RNA-seq and qRT-PCR; this was a normal variation resulting from inherent technical differences between the two types of detection platforms, which are influenced by detection principles, quantification ranges, and normalization strategies. The rate of consistency generally ranges between 80% and 85%, and for low-abundance transcripts, the consistency rate between the two methods decreases further (SEQC/MAQC-III Consortium, 2014; Everaert et al., 2017).

**FIGURE 6:**
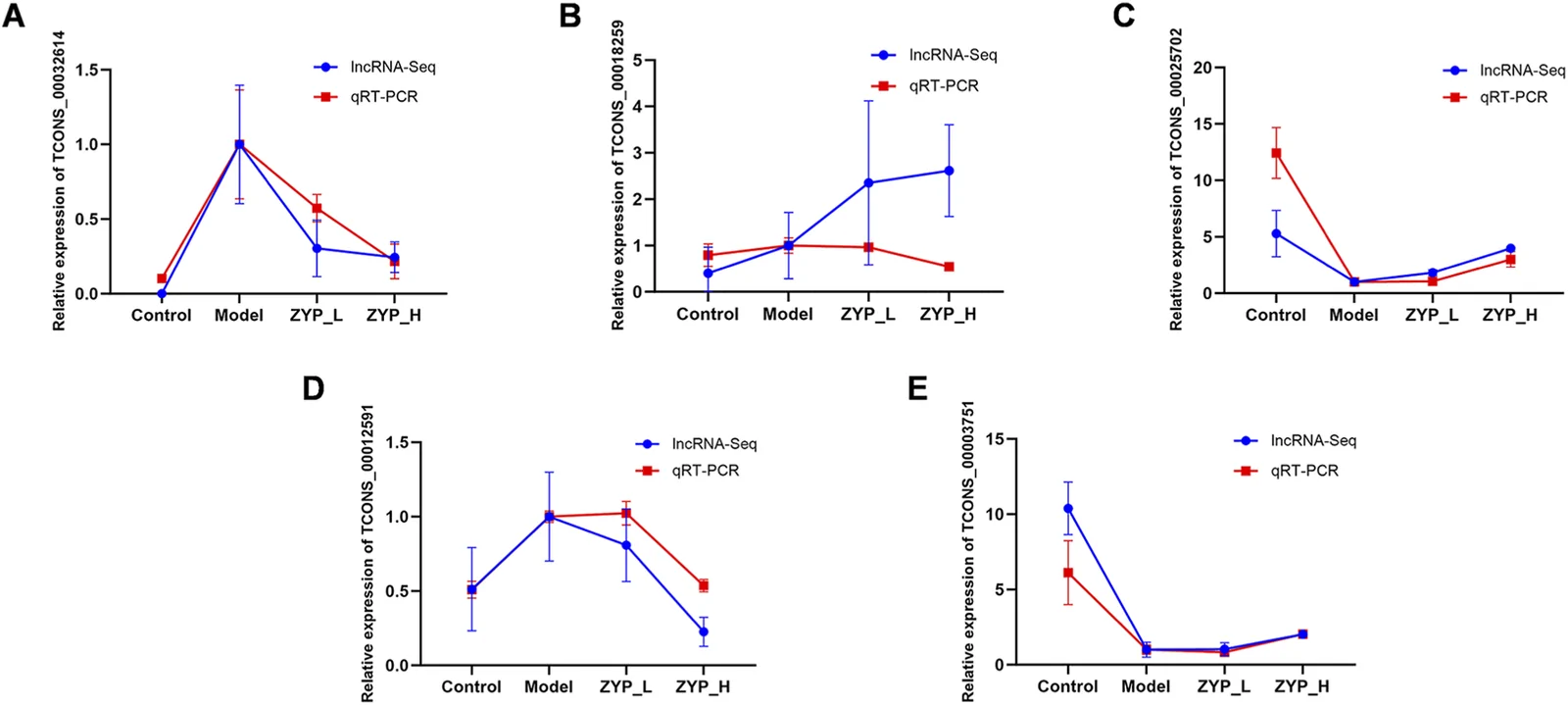
Validation of RNA sequencing results for five target lncRNAs by qRT-PCR. **(A)** TCONS_00032614, **(B)** TCONS_00018259, **(C)** TCONS_00025702, **(D)** TCONS_00012591, **(E)** TCONS_00003751.

Based on qRT-PCR results, the expression pattern of these five lncRNAs, which were suppressed in the disease model and restored after treatment, strongly suggests that they serve as pivotal molecules linking disease damage to drug-mediated repair. Moreover, these lncRNAs are core participants in key signaling pathways associated with cholestasis, further substantiating their regulatory role in cholestasis. Furthermore, for nearly all key lncRNAs, ZYP-H demonstrated a greater degree of improvement than ZYP-L, regardless of whether the analysis was based on lncRNA-Seq or qRT-PCR. This not only demonstrates that ZYP is effective at both high and low doses but also confirms that this efficacy is dose-dependent. TCONS_00018259 exhibited inter-platform technical discrepancies; therefore, it was excluded from the core lncRNA set in subsequent mechanistic analyses to enhance study rigor.

### 3.6 LncRNA-miRNA interaction prediction and network construction

LncRNAs can function as competing endogenous RNAs (ceRNAs), which regulate gene expression by competitively binding to shared miRNAs. Bioinformatic prediction analysis identified that TCONS_00003751 harbored putative binding sites for three miRNAs (rno-miR-328b-3p, rno-miR-328a-3p, and rno-miR-6332), while TCONS_00032614 harbored putative binding sites for two miRNAs (rno-miR-339-5p and rno-miR-149-5p). These candidate pairs exhibited high predicted pairing scores (total score ≥150) and low binding free energies (total energy ≤ −30), rendering them putative candidates for the ceRNA regulatory mechanism.

For the overall lncRNA-miRNA interaction prediction, the top 300 miRNA-lncRNA pairs with the smallest adjusted *p*-values were extracted based on *p*-value ranking. The lncRNA-miRNA interaction network was constructed using the R network package (Butts et al., 2015). In the lncRNA-miRNA interaction network (Figure 7), 10 lncRNAs and 16 miRNAs were identified as common molecular hubs in both the Model vs. Control and ZYP-H vs. Model comparisons. Among these, TCONS_00032614 and rno-miR-149-5p were not only crucial co-regulatory hubs but also formed a paired relationship, suggesting their interaction may play a key regulatory role. Furthermore, most miRNAs targeted distinct lncRNAs across the two comparative groups, suggesting that the same miRNA may mediate divergent biological functions through context-dependent target regulation under diseased versus therapeutic conditions.

**FIGURE 7:**
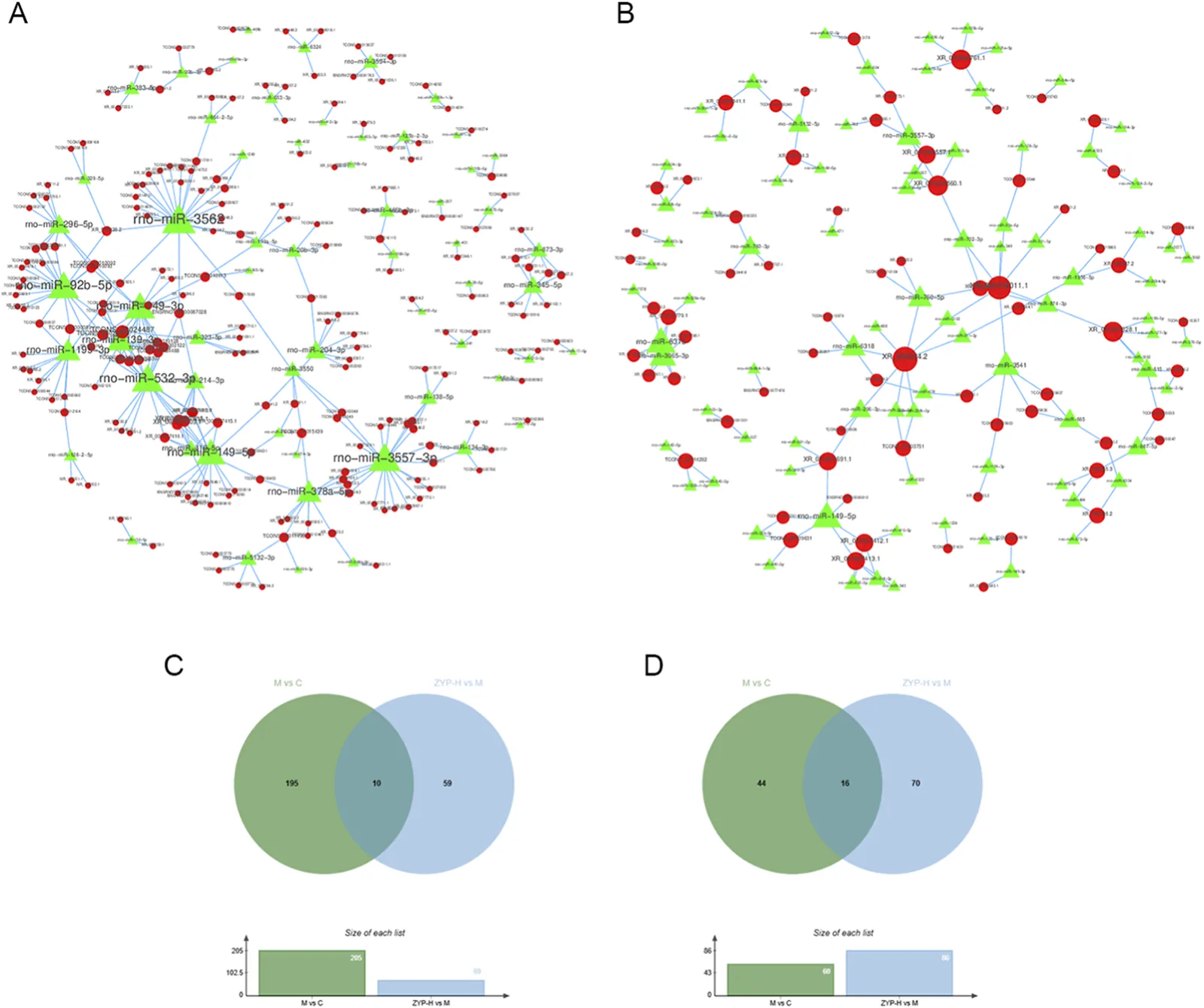
lncRNA–miRNA predicted interaction networks and Venn diagrams. Networks were built using the top 300 significant lncRNA–miRNA pairs under different conditions: **(A)** model vs. control; **(B)** ZYP-H vs. model. Circular nodes denote lncRNAs, triangular nodes denote miRNAs, and node size corresponds to connectivity degree. **(C,D)** Venn diagrams showing overlapping and unique differentially expressed lncRNAs **(C)** and miRNAs **(D)** between the control vs. model and ZYP-H vs. model comparisons.

### 3.7 Screening of candidate lncRNA-miRNA pairs consistent with the ceRNA mechanism

Based on the actual sequencing results, rno-miR-6332 and its corresponding predicted lncRNA-miRNA pairing were excluded due to undetectable miRNA expressions. Expression trend analysis was subsequently performed on the candidate pairs: TCONS_00003751/rno-miR-328b-3p, TCONS_00003751/rno-miR-328a-3p, TCONS_00032614/rno-miR-339-5p, and TCONS_00032614/rno-miR-149-5p (Figure 8). The results showed that, among all candidates, only the following pairs exhibited expression trends consistent with the ceRNA mechanism across all stages: TCONS_00003751/rno-miR-328b-3p, TCONS_00003751/rno-miR-328a-3p, and TCONS_00032614/rno-miR-149-5p.

**FIGURE 8:**
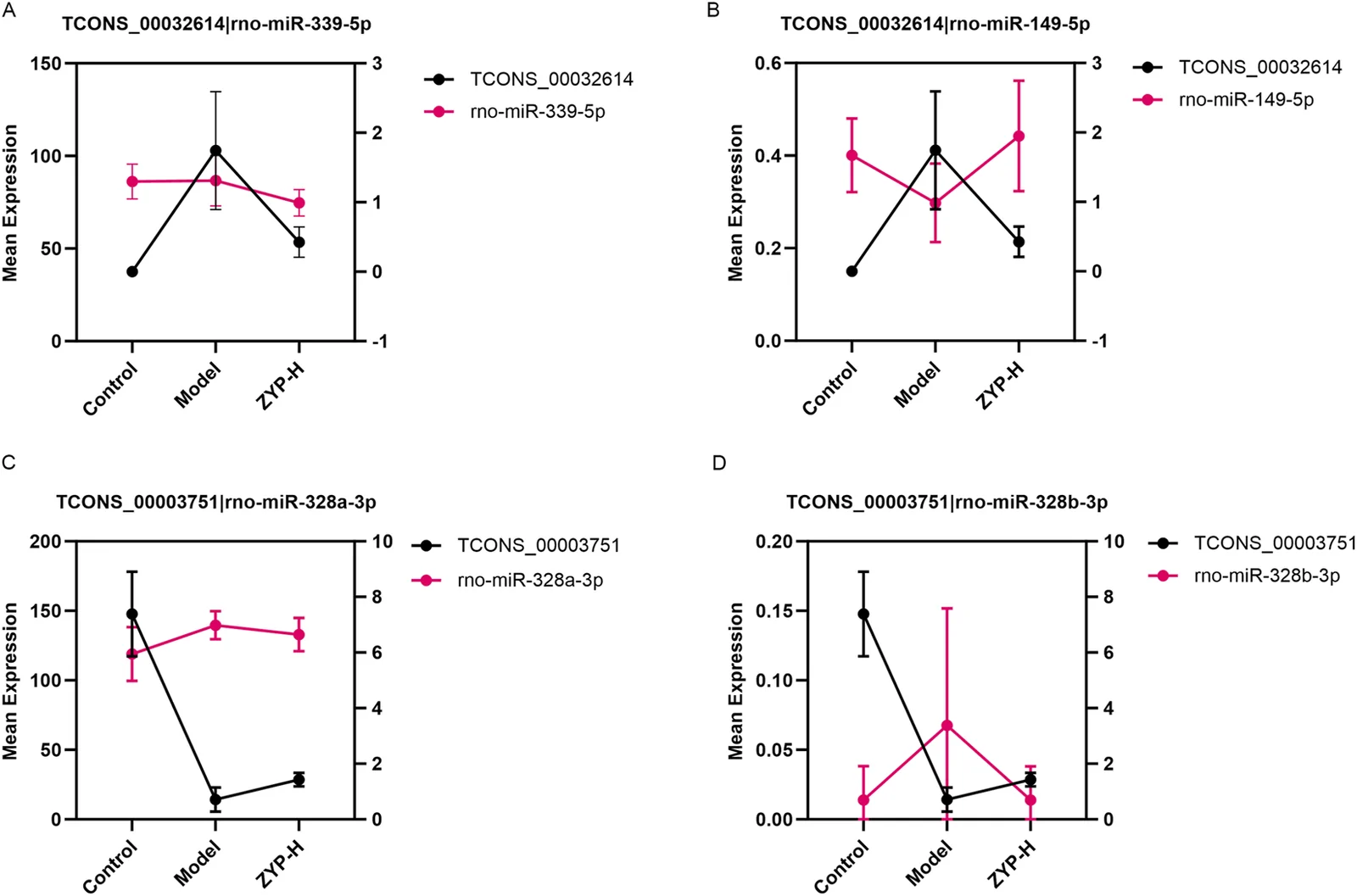
Expression trends of candidate lncRNA–miRNA pairs across control, model, and ZYP-H groups: **(A)** TCONS_00032614/rno-miR-339-5p; **(B)** TCONS_00032614/rno-miR-149-5p; **(C)** TCONS_00003751/rno-miR-328a-3p; **(D)** TCONS_00003751/rno-miR-328b-3p. A dual y-axis layout was adopted for pairs with a substantial discrepancy in the magnitude of expression values: the right y-axis represents the mean expression level of lncRNAs, and the left y-axis represents the mean expression level of corresponding miRNAs Data are shown as mean ± standard deviation (SD).

### 3.8 Screening and quantitative statistics of differentially expressed circRNAs (DEcircRNAs)

As shown in Figure 9A, predicted circRNAs were cross-referenced with established databases. A total of 2,025 circRNAs were both predicted and known circRNAs, which were prioritized for subsequent functional investigation. PCA (Figure 9B) revealed a pronounced separation between the model and control groups, indicating significant transcriptomic disruption induced by cholestasis. The ZYP treatment groups were located between the control and model groups on the PC3 dimension (explaining 9.1% of variance), with limited separation on PC1 and PC2 dimensions, indicating that the overall disease-associated expression pattern had been partially reversed.

**FIGURE 9:**
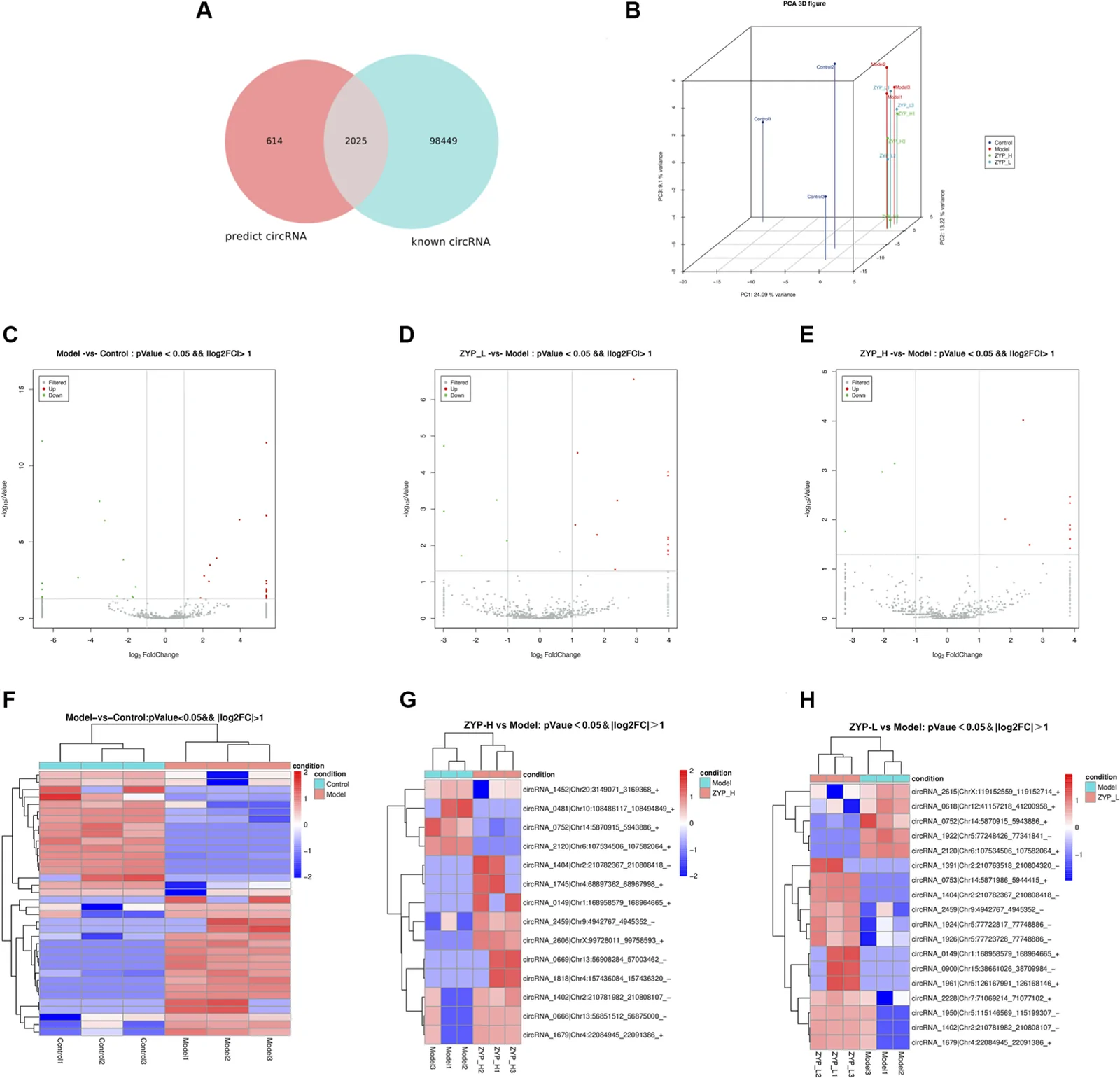
circRNA Sequencing Analysis. **(A)** Venn diagram illustrating the overlap between predicted and known circRNAs. **(B)** PCA plot demonstrating sample clustering patterns and global expression divergence across experimental groups. **(C–E)** Volcano plots visualizing differential circRNA expression. Thresholds were set at |log_2_foldchange| > 1 with p < 0.05. Non-significant genes are shown in gray, significantly upregulated genes in red, and significantly downregulated genes in green. **(F–H)** Hierarchical clustering analysis of differentially expressed lncRNA between experimental groups: **(F)** Control vs. Model, **(G)** ZYP-L vs. Model, and **(H)** ZYP-H vs. Model.

Differentially expressed circRNAs were identified using thresholds of adjusted *p*-value <0.05 and |log_2_foldchange| > 1 (Figures 9C–E). The study identified 36 (19 upregulated, 17 downregulated) DEcircRNAs that were significantly dysregulated following ANIT. ZYP treatment partially reversed these alterations, with the low- and high-dose groups changing 18 (13 upregulated, five downregulated) and 14 (10 upregulated, four downregulated) circRNAs, respectively. Heatmap analysis (Figures 9F–H) further demonstrated ZYP treatment has been demonstrated to exert bidirectional regulatory effects on dysregulated circRNAs. Specifically, it suppressed abnormally elevated circRNAs while restoring pathologically downregulated circRNAs. These findings indicated that ZYP might mitigate cholestasis by reversing circRNA dysregulation.

### 3.9 Function analysis of differentially expressed circRNA

As shown in Figures 10A,B, under ZYP-H treatment, the significantly enriched GO terms for upregulated circRNAs were primarily associated with lipid metabolism and transport, energy metabolism, and stress response processes, whereas downregulated circRNAs were mainly enriched in GO terms related to lipid metabolism and transport, as well as antigen presentation and immune recognition. KEGG pathway analysis (Figures 10C,D) further revealed that circRNAs upregulated by ZYP-H were predominantly involved in cytochrome P450-mediated drug and xenobiotic metabolism pathways, whereas those downregulated by ZYP-H were primarily associated with immune-related pathways, such as the Cell adhesion molecules (CAMs) pathway and Antigen processing and presentation pathways. Following ZYP treatment, upregulated circRNA_1402 and downregulated circRNA_1452, circRNA_0752 were identified as key regulators of these signaling pathways.

**FIGURE 10:**
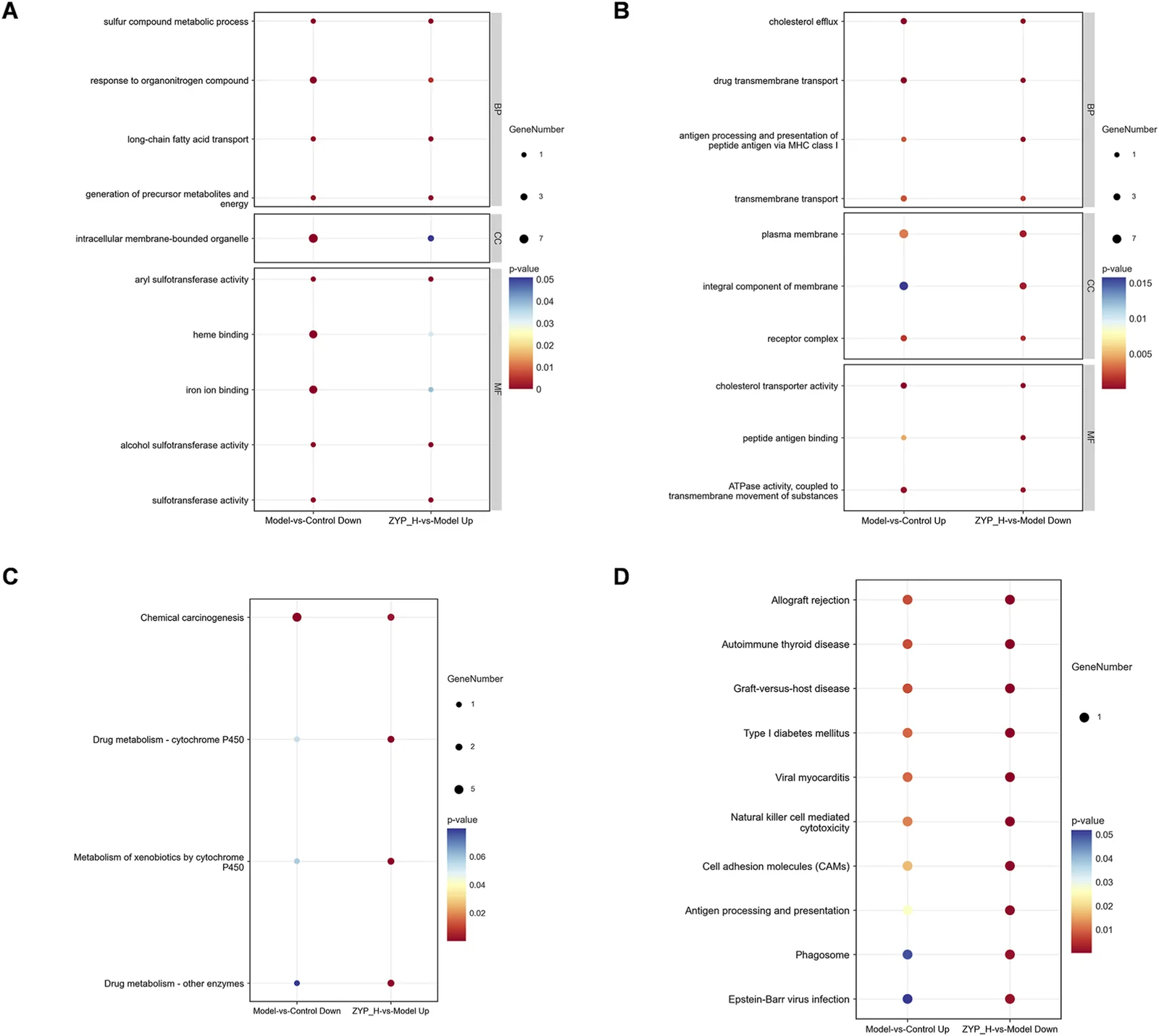
Functional enrichment analysis of DEcircRNAs. GO analysis: **(A)** Top 10 GO terms co-enriched by circRNAs that were downregulated in the model group and upregulated after ZYP treatment. **(B)** Top 10 GO terms co-enriched by circRNAs that were upregulated in the model group and downregulated after ZYP treatment. KEGG analysis: **(C)** All signaling pathways co-enriched by circRNAs that were downregulated in the model group and upregulated after ZYP treatment. **(D)** Top 10 signaling pathways co-enriched by circRNAs that were upregulated in the model group and downregulated after ZYP treatment.

### 3.10 CircRNA-miRNA interaction prediction and network construction

Bioinformatics predictive analysis indicated that circRNA_1402 has potential binding sites with 12 miRNAs (rno-miR-326-3p, rno-miR-330-5p, rno-miR-351-5p, rno-miR-540-3p, rno-miR-423-5p, rno-miR-490-5p, rno-miR-668, rno-miR-3541, rno-miR-3562, rno-miR-702-3p, rno-miR-1306-5p, rno-miR-709), and circRNA_1452 has potential binding sites with six miRNAs (rno-miR-330-5p, rno-miR-23a-5p, rno-miR-361-3p, rno-miR-874-3p,rno-miR-877,rno-miR-3577). All of them had high predicted pairing scores (Total score >150) and low binding free energies (Total energy < −30), rendering them putative miRNA sponges.

For the results of circRNA-miRNA interaction prediction, the top 300 miRNA-circRNA pairs with the smallest *p*-values were extracted. The circRNA-miRNA interaction network (Figure 11) was constructed using the R network package (Butts et al., 2015). Network and Venn diagram results revealed that a total of two circRNAs and 44 miRNAs were identified as common molecular hubs in both the Model versus Control and ZYP-H versus Model comparisons. Among them, circRNA_1452, rno-miR-330-5p, rno-miR-326-3p, rno-miR-23a-5p, and rno-miR-1306-5p were identified as important regulatory targets in both comparisons. Notably, overlapping circRNAs and miRNAs were associated with distinct interaction partners across the two comparisons, reflecting context-dependent network rewiring under pathological versus therapeutic conditions.

**FIGURE 11:**
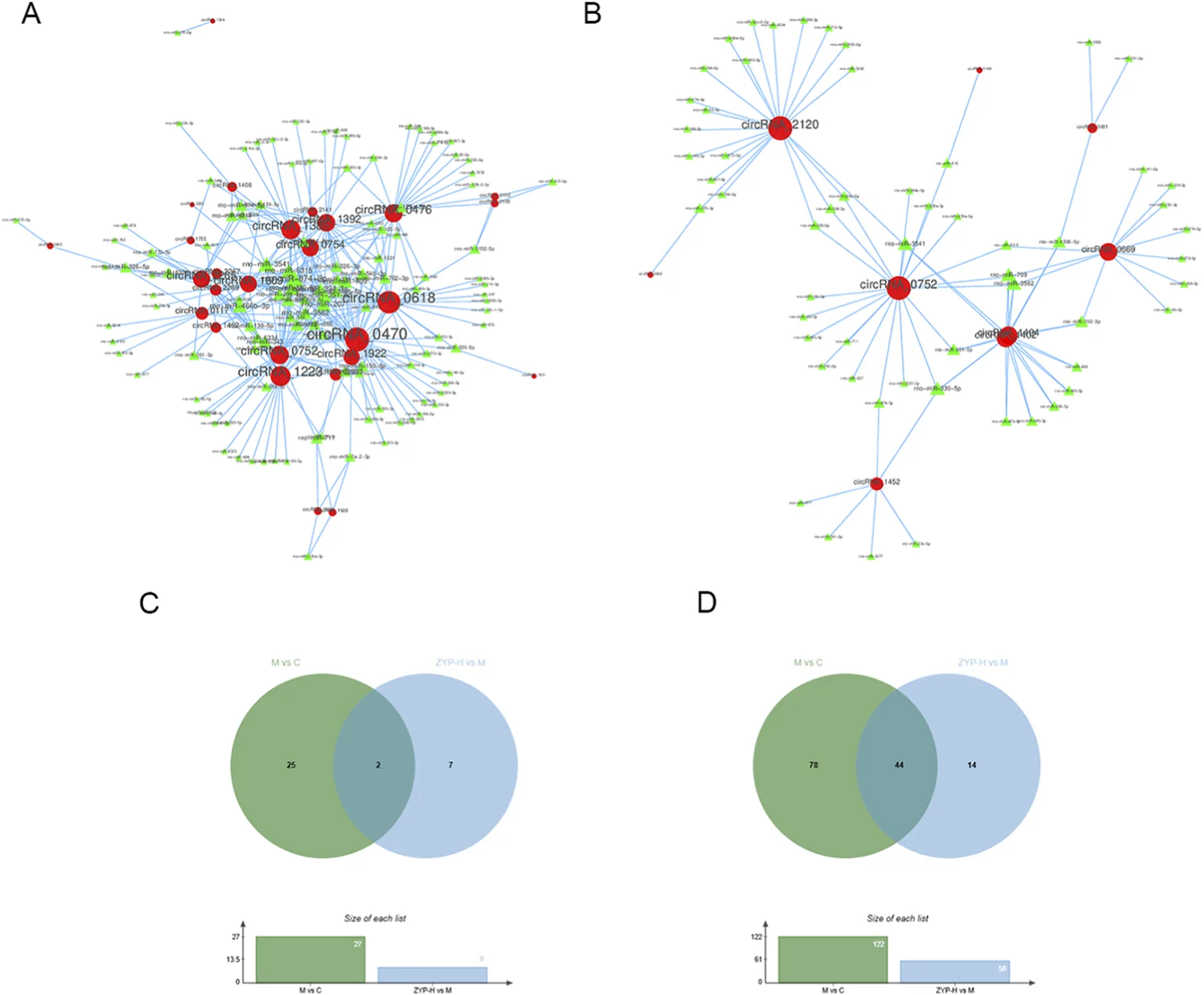
circRNA-miRNA predicted interaction networks and Venn diagrams. Networks were built using the top 300 significant circRNA-miRNA pairs under different conditions: **(A)** model vs. control; **(B)** ZYP-H vs. model. Circular nodes denote circRNAs, triangular nodes denote miRNAs, and node size corresponds to connectivity degree. **(C,D)** Venn diagrams showing overlapping and unique differentially expressed circRNAs **(C)** and miRNAs **(D)** between the control vs. model and ZYP-H vs. model comparisons.

### 3.11 Screening of candidate circRNA-miRNA pairs consistent with the ceRNA mechanism

After excluding undetectable miRNAs (rno-miR-668, rno-miR-3541, rno-miR-3562, and rno-miR-709) and their associated predicted pairings, expression trend analysis was performed on the remaining predicted circRNA-miRNA pairs (Figures 12A–F, 13A–F). Both the rno-miR-1306-5p/circRNA_1402 (Figure 12B) and rno-miR-23a-5p/circRNA_1452 pairs (Figure 13B) exhibited expression dynamics consistent with the characteristic expression patterns of the ceRNA mechanism, suggesting that they might be candidate ceRNA pairs.

**FIGURE 12:**
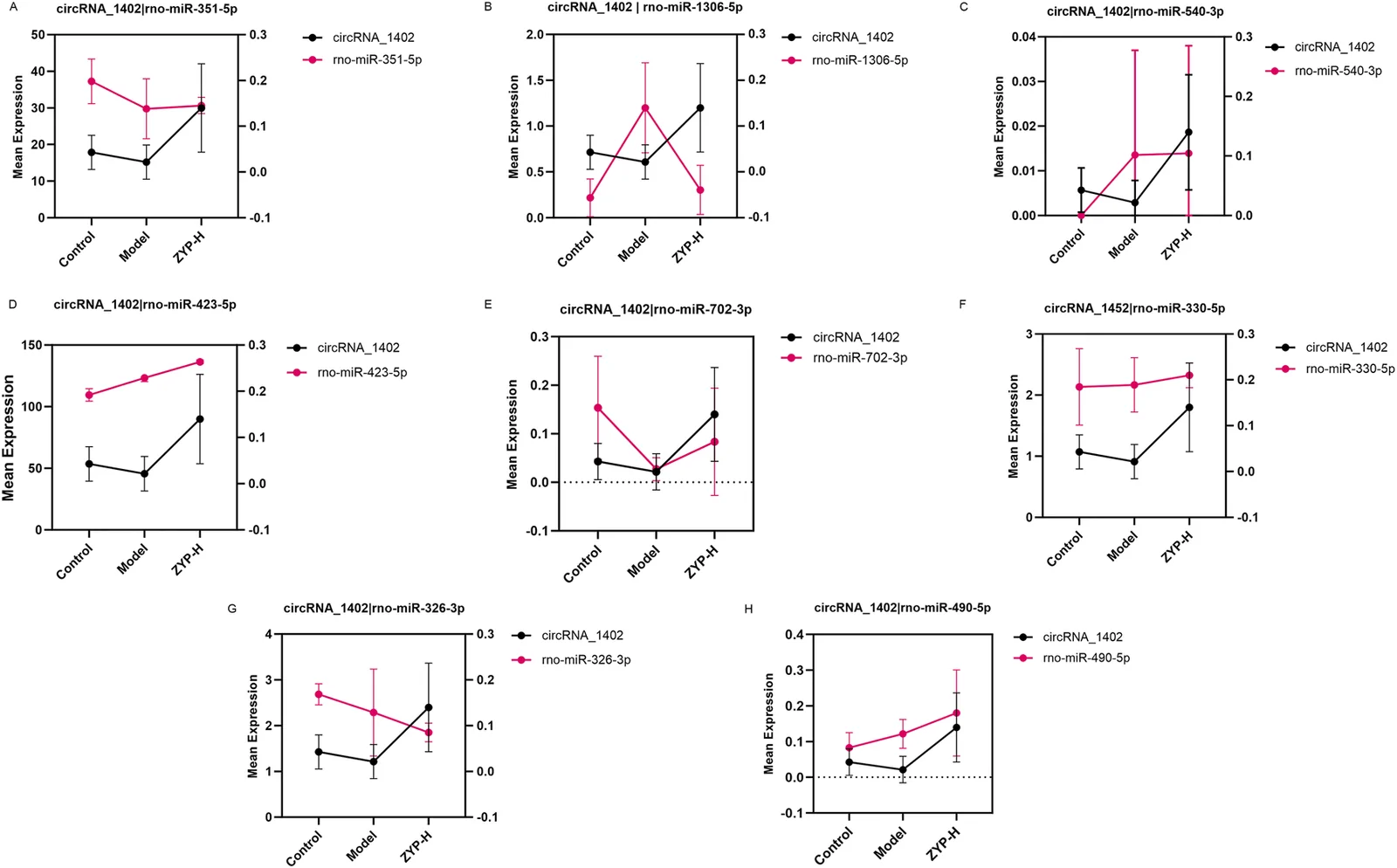
Expression trends of circRNA_1402 and its predicted target miRNAs in control, model, and ZYP-H groups. A dual y-axis layout was adopted for pairs with a substantial discrepancy in the magnitude of expression values: the right y-axis represents the mean expression level of circRNAs, and the left y-axis represents the mean expression level of corresponding miRNAs. **(A)** circRNA_1402 and rno-miR-351-5p; **(B)** circRNA_1402 and rno-miR-1306-5p; **(C)** circRNA_1402 and rno-miR-540-3p; **(D)** circRNA_1402 and rno-miR-423-5p; **(E)** circRNA_1402 and rno-miR-702-3p; **(F)** circRNA_1452 and rno-miR-330-5p; **(G)** circRNA_1402 and rno-miR-326-3p; **(H)** circRNA_1402 and rno-miR-490-5p. Data are shown as mean ± standard deviation (SD).

**FIGURE 13:**
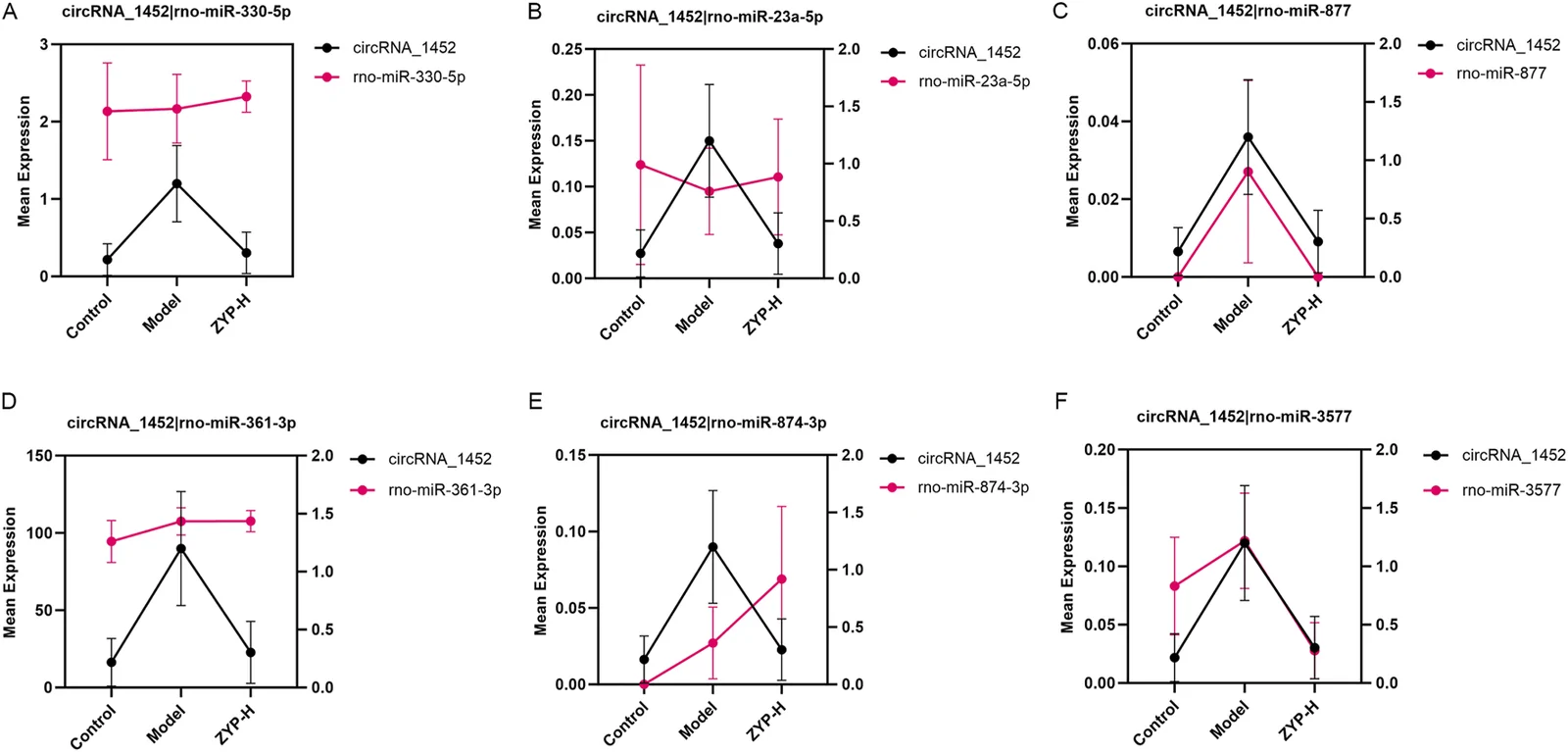
Expression trends of circRNA_1452 and its predicted target miRNAs in control, model, and ZYP-H groups. A dual y-axis layout was adopted for pairs with a substantial discrepancy in the magnitude of expression values: the right y-axis represents the mean expression level of circRNAs, and the left y-axis represents the mean expression level of corresponding miRNAs. **(A)** circRNA_1452 and rno-miR-330-5p; **(B)** circRNA_1452 and rno-miR-23a-5p; **(C)** circRNA_1452 and rno-miR-877; **(D)** circRNA_1452 and rno-miR-361-3p; **(E)** circRNA_1452 and rno-miR-874-3p; **(F)** circRNA_1452 and rno-miR-3577. Data are shown as mean ± standard deviation (SD).

## 4 Discussion

In recent years, the role of lncRNAs and circRNAs in the pathogenesis and treatment of cholestasis has attracted significant attention. By integrating animal experimental results with bioinformatics analysis, this study first revealed the key molecular mechanism by which ZYP significantly alleviates cholestasis through regulating lncRNAs and circRNAs. These findings not only provided new theoretical support for the clinical application of ZYP but also proposed novel intervention strategies for targeted therapy of cholestasis.

ZYP treatment significantly reduced the ANIT-induced elevations of serum biochemical parameters (DBIL, TBA, TC, and TG) and liver injury markers (ALT, AST, ALP, and γ-GT). Moreover, the improvement was more pronounced in the ZYP-H group than in the ZYP-L group. This suggested that ZYP-H ameliorated the pathological state of cholestasis in a dose-dependent manner. Concurrently, light microscopic pathological examination of liver tissue revealed that ZYP treatment mitigated inflammatory cell infiltration induced by ANIT and preserved liver tissue architecture. These findings were consistent with those of earlier studies (Han et al., 2023). The study further explored the prediction of miRNA targets for lncRNAs and circRNAs, as well as their expression trends. A marked separation was observed in the PCA score plot after ANIT administration. By contrast, ZYP caused the overall expression profiles of lncRNAs and circRNAs to shift back toward those of the control group. This suggested that the anti-cholestatic effect of ZYP was associated with alterations of lncRNAs and circRNAs. Furthermore, the study conducted a preliminary investigation into the mechanism by which ZYP exerts its anti-cholestatic effects through the regulation of lncRNAs and circRNAs. This was achieved by analyzing the signaling pathways enriched in DElncRNAs and DEcircRNAs, together with the construction of target prediction networks and expression trend plots between lncRNAs/circRNAs and miRNAs.

### 4.1 Analysis of possible mechanisms of lncRNA

#### 4.1.1 Enrichment analysis suggests potential mechanisms

Cholestasis not only impairs the digestion and absorption of fats and fat-soluble vitamins (de Aguiar Vallim et al., 2013) but also leads to abnormal lipid levels (Charlat et al., 2025). Patients with PBC and idiopathic cholestatic disease typically exhibit increased blood lipid levels (Reshetnyak and Maev, 2022; Zhan et al., 2022). With respect to lipid metabolism, cholesterol serves as the primary precursor for BAs and is essential for the formation and flow of bile (Lu et al., 2025; Xu et al., 2025). In summary, cholestasis is closely associated with lipid metabolism disorders. The accumulation of toxic BAs induces apoptosis, disrupts membrane integrity, and releases cellular components. These injuries activate immune cells through damage-associated molecular patterns (DAMPs), initiating the inflammatory cascade (Chen and Zhang, 2023). Dysregulation of the gut-liver axis further exacerbates inflammatory damage (Blesl and Stadlbauer, 2021). Immune and inflammatory responses also interact with lipid metabolism dysfunction. During cholestasis, hepatic lipid accumulation triggers compensatory mitochondrial fatty acid β-oxidation, promoting reactive oxygen species (ROS) production. Excessive ROS further activates inflammatory pathways while inhibiting β-oxidation, leading to exacerbated lipid accumulation (Jin et al., 2020). Therefore, this study prioritized the analysis of lncRNA alterations in key lipid metabolism pathways such as arachidonic acid metabolism, adipocytokine signaling, and bile secretion, while also examining lncRNA changes in inflammation-related pathways, including MAPK, TNF, NF-κB, and inflammatory bowel disease (IBD) pathway.

By screening regulatory lncRNAs in those pathways, five potential therapeutic lncRNA targets were identified: TCONS_00032614, TCONS_00025702, TCONS_00003751, TCONS_00018259, and TCONS_00012591. With the exception of TCONS_00018259, the qRT-PCR validation results were generally consistent with the RNA-seq data, supporting the reliability of our transcriptomic expression profiling.

##### 4.1.1.1 Lipid metabolism-related signaling pathways

In the adipokine signaling pathway, both upregulated and downregulated DElncRNAs were enriched, with the upregulated lncRNA TCONS_00018259 and the downregulated TCONS_00012591 being particularly prominent. This suggested that ZYP-H may regulate different segments of the adipokine signaling pathway through a multi-target and precise mechanism. Classic adipokines primarily include adiponectin and leptin. Adiponectin has anti-inflammatory and anti-fibrotic effects; it is compensatorily elevated during cholestasis to mitigate hepatic injury (Tacke et al., 2005). Serum leptin levels are increased in cholestasis (Breidert et al., 2004; Rieger et al., 2005), promoting Akt phosphorylation, thereby exacerbating cholangiocyte responses, inflammation, and the fibrotic process (La Cava and Matarese, 2004; Petrescu et al., 2022). Additionally, the upregulated TCONS_00025702 acted on the arachidonic acid metabolism pathway. Previous studies have found that dietary AA supplementation significantly reduces cholestasis-related serum markers (ALT, AST, ALP, TBA, and TBIL) and decreases bile duct proliferation and the degree of cholestasis (Ma et al., 2024). Metabolites of AA, such as epoxyeicosatrienoic acids (EETs), exhibit anti-inflammatory and anti-fibrotic effects, and promote bile acid excretion by activating PPAR. Furthermore, the upregulated TCONS_00003751 acted on the bile secretion pathway (Christmas, 2015; Kaspera and Totah, 2009; Shi et al., 2022). Our previous research had also demonstrated that the miRNA-enriched signaling pathways upregulated by ZYP-H involved bile secretion (Han et al., 2023). This experiment further corroborated that finding from the perspective of lncRNA. The functional state of the bile secretion pathway directly determines the efficiency of bile excretion and the digestion and absorption of fats (Marin et al., 2015). Therefore, dysfunction of this pathway represents a key contributor to cholestasis. Integrating previous and current findings, it is hypothesized that ZYP modulates the key lncRNAs TCONS_00012591, TCONS_00018259, and TCONS_00003751 to achieve a triple therapeutic impact: rebalancing adipokine secretion, boosting beneficial arachidonic acid metabolites, and directly regulating bile secretion. This multifaceted synergistic action exerts anti-inflammatory, anti-fibrotic, and bile excretion-promoting effects, ultimately alleviating cholestatic liver injury.

##### 4.1.1.2 Inflammation-related signaling pathways

Significant changes in lncRNA expression were observed across multiple key inflammation-related signaling pathways following ZYP-H intervention. In the IBD pathway, downregulated DElncRNAs showed significant enrichment, including TCONS_00032614. In the NF-κB, MAPK, and TNF signaling pathways, the expression profile of lncRNAs exhibited bidirectionality (both upregulation and downregulation), suggesting that ZYP-H may precisely modulate key components of these pathways through a multi-target synergistic mechanism.

In the pathogenesis of cholestasis, MAPK, TNF, and NF-κB signaling pathways all play critical roles and exhibit a dual functional nature. The NF-κB signaling pathway promotes inflammatory responses, yet activation of certain branches can inhibit hepatocyte apoptosis mediated by Fas and TNFR1 death receptors, contributing to a protective effect (Miyoshi et al., 2001). The MAPK signaling pathway can exacerbate cholestasis by inducing the endocytosis of transporters (Bsep and Mrp2) (Toledo et al., 2017). Moreover, while its JNK branch mediates apoptosis through phosphorylation of mitochondrial Bcl-2/Bax family proteins (Uehara et al., 2004), its ERK1/2 branch reduces apoptosis rates by activating pro-survival proteins and inhibiting pro-death proteins (Cook et al., 2017). The TNF pathway exhibits functional duality by promoting and counteracting apoptosis. On one hand, it can trigger pro-apoptotic effects by releasing ROS, cytochrome, and Bax proteins through TNFR1 or mitochondria, thereby initiating the caspase cascade (Aggarwal et al., 2012). On the other hand, this pathway can upregulate anti-apoptotic genes such as TRAF1/2, IAPs, and Bcl-xl (Gozzelino et al., 2008). Furthermore, the TNF pathway disrupts farnesoid X receptor (FXR)-mediated bile acid homeostasis and promotes hepatic fibrosis (Abdulaal et al., 2024). The MAPK, TNF, and NF-κB pathways engage in complex interactions and cross-regulation. First, the TNF pathway serves as a critical “initiator” for both the MAPK and NF-κB pathways (Kim and Choi, 2010; Napetschnig and Wu, 2013; Rieger et al., 2005). Second, NF-κB and JNK counterbalance each other; activation of NF-κB can suppress sustained JNK activation, thereby preventing excessive JNK-mediated apoptosis (Guo et al., 2024). The cross-pathway regulatory potential of TCONS_00018259 and TCONS_00012591 in these inflammatory pathways indicated that ZYP may not merely modulate downstream effectors of a single pathway. Instead, it is more likely to act on key upstream hubs or potentially regulate critical nodal points of crosstalk, such as the inhibitory effect of NF-κB on JNK.

IBD pathways are also closely associated with cholestasis. BAs can activate the FXR through the enterohepatic circulation, negatively regulating BA synthesis (Meadows et al., 2020; Sayin et al., 2013). Additionally, gut bacterial antigens may translocate to the liver via the portal vein, disrupting the tight junction barrier between hepatocytes and leading to bile duct injury and exacerbating cholestasis (O'Toole et al., 2012). Furthermore, previous studies have shown that colitis may suppress bile acid synthesis by activating NF-κB signaling pathway, thereby alleviating liver injury and fibrosis (Gui et al., 2023). This finding points to a collaborative role of the IBD and NF-κB pathways in the regulation of cholestasis.

Based on the above evidence, we hypothesize that ZYP-H achieves integrated regulation of multiple inflammatory pathways by modulating the expression of key lncRNAs. It suppresses excessive inflammatory responses in the NF-κB, MAPK, and TNF signaling pathways, mitigates apoptosis mediated by these pathways, and enhances the function of their protective branches. Additionally, IBD pathway also participates in this regulatory network via the gut-liver axis. Through such multi-targeted and multi-level synergistic mechanisms, ZYP-H collectively contributes to the amelioration of cholestatic pathology.

#### 4.1.2 Analysis of candidate lncRNA-miRNA pairs consistent with the ceRNA mechanism

Through bioinformatics analysis, the study identified two lncRNAs and their corresponding high-affinity miRNA pairs, whose expression trends align with the ceRNA regulatory mechanism: TCONS_00003751 (upregulated) paired with rno-miR-328b-3p and rno-miR-328a-3p (both downregulated), and TCONS_00032614 (downregulated) paired with rno-miR-149-5p (upregulated).

Based on existing research, miR-328-3p has been shown to exhibit pro-injury and pro-transformation effects in various disease models: in alveolar epithelial cell acute injury, elevated miR-328-3p promotes apoptosis by suppressing Creb1 (Li Z. et al., 2023); in head and neck squamous cell carcinoma, upregulation of miR-328-3p promotes epithelial-mesenchymal transition (EMT) (Ma et al., 2021); and in ovarian cancer stem cells, miR-328-3p maintains stemness characteristics (Srivastava et al., 2019). Notably, key pathological processes in cholestasis, including hepatocyte apoptosis (Qin et al., 2020), cholangiocyte injury accompanied by EMT-like phenotypic changes (Strazzabosco and Fabris, 2011), and aberrant stem cell activation-driven fibrogenesis (Carpino et al., 2019; Sun et al., 2016). Regarding miR-149-5p, previous studies have shown that in bronchial epithelial cells, it targets Table 2 to suppress the MAPK/NF-κB signaling pathway, thereby attenuating pollutant-induced inflammation (Li Q. et al., 2023); in vascular endothelial cells, it directly targets TNF-α to alleviate high glucose-induced endoplasmic reticulum stress (Yuan et al., 2017). In cholestasis, bile acid accumulation activates the NF-κB inflammatory signaling pathway and induces endoplasmic reticulum stress in both hepatocytes and cholangiocytes (Qin et al., 2006; Har-Zahav et al., 2026).

Although these findings were obtained from non-cholestatic models, the cellular phenotypic alterations involved and core signaling pathways are highly conserved across diverse cellular and pathological contexts. Accordingly, we propose a putative ceRNA regulatory hypothesis: upregulated TCONS_00003751 may serve as a ceRNA sponge for miR-328a-3p and miR-328b-3p, and thus participate in modulating hepatobiliary structural injury and remodeling during cholestasis. Meanwhile, downregulated TCONS_00032614 may reduce its sequestration of miR-149-5p, which may in turn strengthen the miRNA’s protective effects against inflammatory injury and cellular stress. Nevertheless, these inferences are limited by cross-model extrapolation and lack of direct molecular interaction evidence, and thus should be interpreted with caution until validated in ANIT-induced cholestasis and ZYP treatment settings.

### 4.2 Analysis of potential mechanisms of circRNA

#### 4.2.1 Enrichment analysis reveals underlying mechanisms

KEGG pathway analysis revealed that after ZYP-H treatment, upregulated circRNAs were primarily enriched in the “Cytochrome P450 metabolism of xenobiotics” pathway, while downregulated circRNAs were mainly enriched in the “Cell adhesion molecules (CAMs) “ and “Antigen processing and presentation” pathways. Specifically, circRNA_1402 was identified as a key regulator of the “Cytochrome P450 metabolism of xenobiotics” pathway, whereas circRNA_1452 was found to regulate both the “CAMs”and “Antigen processing and presentation” pathways.

The “Cytochrome P450 metabolism of xenobiotics” pathway encompasses various toxic substances closely associated with cholestasis, such as Benzo [a]pyrene (B [a]P), Aflatoxin B1 (AFB1), bromobenzene, and 1-nitronaphthalene (1-NN). Research has shown that active metabolites of B [a]P increase the risk of cholangiocarcinoma (Kirby et al., 1995); AFB1 can promote bile acid synthesis while inhibiting its excretion (Thompson and Strautnieks, 2001; Yu et al., 2024; Jia et al., 2021); bromobenzene damages liver acinar zone 3, reduces basal bile flow, and leads to impaired bile movement (Shamir et al., 1993); and 1-NN has been confirmed to induce hepatotoxicity (Verschoyle et al., 1993). Cholestasis causes the accumulation of these substances in the liver, while simultaneously weakening the liver’s detoxification capacity, thereby exacerbating both cholestasis and the extent of hepatocyte damage. Therefore, we hypothesize that ZYP protects against cholestatic injury by upregulating circRNA_1402, thereby enhancing CYP450-mediated metabolism and clearance of these toxic xenobiotics.

Regarding the “CAMs” and “Antigen processing and presentation” pathways, genome-wide association studies (GWAS) have confirmed their significant association with PBC (Kar et al., 2013). Multiple adhesion molecules are upregulated in damaged bile ducts, portal areas, and hepatic parenchyma during liver tissue injury, promoting inflammatory cell extravasation and epithelial migration. Dendritic cells positively respond to these immune molecules, promoting antigen presentation and CD4^+^ helper T cell activation, thereby triggering autoimmune responses (Nakanuma et al., 1997). Consequently, ZYP’s therapeutic effect on cholestasis may stem from its ability to regulate “CAMs” and “antigen processing and presentation” by downregulating circRNA_1452, thereby inhibiting immune-mediated damage.

#### 4.2.2 Analysis of candidate circRNA-miRNA pairs consistent with the ceRNA mechanism

This study performed expression trend analysis between the screened circRNAs and their predicted target miRNAs with high binding affinity, identifying circRNA–miRNA pairings with potential ceRNA relationships: rno-miR-1306-5p (downregulated)/circRNA_1402 (upregulated) and rno-miR-23a-5p (upregulated)/circRNA_1452 (downregulated).

Existing studies have shown that, in bronchial epithelial cell models, overexpression of miR-23a-5p inhibits ROS accumulation and MAPK signaling pathway activation, and reduces the secretion of inflammatory factors (Chang et al., 2024). In fibroblast-like synoviocytes, overexpression of miR-23a-5p simultaneously inhibits TNF-α-induced inflammatory damage and TLR4/NF-κB signaling activation by targeting TLR4, thereby promoting apoptosis of inflammatory cells (Bao et al., 2021). Additionally, berberine (BBR, one of the main components of ZYP) has been reported to upregulate miR-23a, thereby suppressing TLR4/TRAF2-mediated ASK1/p38 phosphorylation and attenuating LPS/TNF-α-induced macrophage inflammatory responses, with concomitant reduction of TNF-α, IL-6, and IL-23 levels (Sujitha et al., 2018). Based on these findings, it can be inferred that downregulated circRNA_145 may act as a molecular sponge, binding to and inhibiting miR-23a-5p, thereby exerting an anti-inflammatory effect on cholestasis.

Regarding the rno-miR-1306-5p/circRNA_1402 axis, in LPS-induced intestinal epithelial cell injury models, inhibition of miR-1306-5p upregulates HIPK2 expression, which in turn decreases cell apoptosis and promotes cell survival (Feng et al., 2024; Zhang et al., 2026). In renal ischemia-reperfusion injury, renal tubular epithelial cells (RTECs) release exosomes carrying miR-1306-5p; upon uptake by macrophages, these exosomes drive M2 macrophage polarization and accelerate interstitial fibrosis (Tang et al., 2025). These findings indicate that miR-1306-5p exerts pro-apoptotic and pro-fibrotic effects. Based on a combination of previous studies and the blinding prediction and reverse expression trends identified in this study, we hypothesize that upregulated circRNA_1402 may function as a potential molecular sponge to adsorb and inhibit miR-1306-5p, thereby alleviating apoptosis and fibrosis induced by cholestasis.

Based on the existing studies, it is reasonable to infer that ZYP-H may alleviate cholestasis-induced inflammatory injury, apoptosis, and fibrosis via regulating the circRNA_1402/rno-miR-1306-5p and circRNA_1452/rno-miR-23a-5p axes. It should be noted that these findings do not originate directly from an ANIT-induced cholestasis model and require further validation by functional experiments.

### 4.3 Limitations of research

This study confirmed the ameliorative effect of ZYP on cholestasis through histopathological examination and liver function-related serological tests, and preliminarily explored its potential mechanisms based on enrichment analysis, miRNA binding site prediction and network construction for lncRNAs and circRNAs, as well as sequencing-based expression trend analysis. However, several limitations remain to be addressed. First, there was a lack of target validation. The qRT-PCR experiments were not conducted to validate expression changes in key miRNAs that successfully paired with lncRNA and circRNA. Second, functional experiments were absent. The specific mechanism of ZYP was not further elucidated through functional overexpression or knockdown experiments combined with the detection of known bile acid regulatory targets, inflammatory factors, and lipid metabolism-related indicators. Third, there was no direct evidence of molecular interactions. Techniques such as dual luciferase reporter assays, RNA immunoprecipitation (RIP), or RNA pull-down were not employed to directly validate the interaction between lncRNA/circRNA and miRNA. Future studies should refine these experiments, construct a lncRNA/circRNA-miRNA-mRNA ceRNA regulatory network, and further validate the proposed mechanisms suggested in this research.

## 5 Conclusion

This study demonstrated that ZYP significantly alleviated cholestasis in a dose-dependent manner. This therapeutic effect may be associated with the modulation of lncRNAs and circRNAs involved in lipid metabolism and inflammatory pathways. On this basis, several candidate ceRNA regulatory axes were preliminarily screened through multidimensional cross-validation combining target binding prediction and bidirectional expression trend constraint. These axes represent testable mechanistic hypotheses underlying the protective effect of ZYP, and further functional experiments are required to verify their specific roles. Collectively, these findings offer preliminary mechanistic insights into the application of traditional Chinese medicine for cholestasis management, and lay a foundation for subsequent in-depth mechanism research.

## Data Availability

The datasets presented in this study can be found in online repositories. The names of the repository/repositories and accession number(s) can be found below: https://www.ncbi.nlm.nih.gov/, PRJNA1072646.
