# Supplementary material for: Zhuyu pill relieves cholestasis in rats by modulating the expression of lncRNAs and circRNAs
Source: Front Pharmacol. 2026 Sep 29;17:1744237. doi: 10.3389/fphar.2026.1744237 (PMC13658601; doi:10.3389/fphar.2026.1744237)
Supplement: Supplementary file 2 [file DataSheet2.pdf]

ConPhyMP checklist of items for conducting and reporting analytical methods[1,2] relevant for extract type A (for species or botanical drugs covered in a monograph in one of the national or regional pharmacopoeias)

SECTION/TOPIC	ITEM NO.	CHECKLIST ITEM	YES	NO	NOT APPLICABLE	PAGE NO., IF ANY
Type of extract	1	A – Confirm that the species or botanical drug under investigation is covered in a monograph in one of the national or regional pharmacopoeias.	✓			In the secor
Preferred/main methods for extract characterisation/ chemical analysis	2	Compliance with pharmacopoeial standards to be followed:				
		(a) The description of the active ingredients in the botanical drug (if known) or analytical marker compounds as defined.	✓			d paragraph
		(b) An analysis as defined in the monograph is needed if the extract has not been supplied with a certificate.		✓		
		(c) If the preparation was purchased, the manufacturer and certificate of analysis need to be included.			✓	
		Including either the preferred or alternative approaches for characterisation:				
		(a) Triple chemical fingerprinting methods, each with one or more detection parameters.		✓		
		(b) Quantification of at least two marker compounds (unless this is not feasible, evidence needs to be provided), and justification of the choice of markers (if applicable).	✓			In the secor
Alternative methods for extract characterisation/chemical analysis	3	(a) Single chemical fingerprinting method with at least three different detection parameters (i.e., altered detection parameters, like TLC/ HPTLC with different staining reagents and/or UV excitation wave-lengths, HPLC-DAD/LCDAD with different wavelengths). The same applies to coupling MS or NMR to chromatographic techniques.		✓		
		(b) Quantification of at least two marker compounds (unless this is not feasible, evidence needs to be provided), and justification of the choice of markers (if applicable).	✓			osection 2.2
Use of reference standards	4	(a) Direct overlay of the chromatogram of the sample with that of an officially specified reference standard (if applicable). (b) Chromatographic fingerprinting: Direct overlay of the chromato-gram of the sample with that of official reference standards of the powdered plant material or the dry extract from the plant material.		✓		
Comparison of different extracts/ samples of the same plants	5	(a) Direct comparison of the chromatographic/spectroscopic system and/or scoring system for "similarity" to be followed.			✓	

Note: Please also include here the following information about your submitted manuscript:

Name of the journal:	Frontiers in Pharmacology - Ethnopharmacology
Date of the enquiry:	2026-07-12
Title of the manuscript:	Zhuyu Pill relieves cholestasis in rats by modulating
List of the authors:	Rui Cheng, Zhili Zheng, Zongying Xu, Jun Han, Han Yu

[1] Please acknowledge/cite this as follows: Heinrich M, Jalil B, Abdel-Tawab M, Echeverria J, Kulić Ž, McGaw LJ, et al. Best Practice in the chemical characterisation of extracts used in pharmacological and toxicological research—The ConPhyMP—Guidelines. Frontiers in Pharmacology. 2022;13:953205. https://doi.org/10.3389/fphar.2022.953205

[2] We strongly recommend reading this checklist in conjuncton with ConPhyMP 2022 explanaton and elaboraton for important clarifications on all items. If relevant, we also recommend after reading Heinrich et al. (2020) Best practice in research—Overcoming common challenges in phytopharmacological research. Journal of Ethnopharmacology. 2020;246:112230. https://doi.org/10.1016/j.jep.2019.112230
