# Supplementary material for: Zhuyu pill relieves cholestasis in rats by modulating the expression of lncRNAs and circRNAs
Source: Front Pharmacol. 2026 Sep 29;17:1744237. doi: 10.3389/fphar.2026.1744237 (PMC13658601; doi:10.3389/fphar.2026.1744237)
Supplement: Supplementary file 3 [file Supplementaryfile1.docx]

Supplementary Material

# 1 Supplementary Note

# 1.1 Supplementary Note S1 *ZYP Preparation and Quality Control*

In this study, the ZYP decoction was prepared by boiling the herbs in water twice. For every 240 ml of water, there were 6 g each of Coptis chinensis Franch. and Tetradium ruticarpum (A. Jussieu) T. G. Hartley. The mixture was heated at 100°C for 45 min, filtered, and the suspension was collected for later use. The filtered crude herbs were then added to another 240 ml of water, heated at 100°C for 45 min, filtered, and the suspension was collected. The two suspensions were mixed thoroughly and concentrated to 120 ml using a rotary evaporator. As a result, 120 ml of solution was extracted from 12 g of crude Chinese herbs, making the final concentration of ZYP 0.1 g/ml (w/v). The extraction solution was stored at -20°C before administration.

Four alkaloids in ZYP (berberine, coptisine, evodiamine, and rutecarpine) were analyzed by HPLC using an Agilent 1,260 Infinity II (Agilent Technologies Inc., California, United States). For chromatographic separation, a Welch Ultimate XB-C18 Column (4.6 mm × 250 mm, 5 μm, Maryland, United States) was employed at a column temperature of 30°C. The linear gradient mobile phase was composed of mobile phase A (50 mM monopotassium phosphate +0.4% sodium heptane sulfonate, pH = 4) and mobile phase B (pure methanol). A gradient elution was employed (0–15 min, 95% A, 5% B; 15–40 min, 50% A, 50% B; 40–55 min, 30% A, 70% B; 55–60 min, 95% A, 5% B) at a flow rate of 1.0 ml/min with a 10 μL injection volume. Detection wavelengths were set as follows: (0-44 min, 345 nm; 44-48 min, 226 nm; 48-60 min, 345 nm).

**1.2 Supplementary Note S2: *LncRNA Screening and Prediction***

(1). The merged transcripts are systematically compared to reference transcripts using Cuffcompare (Ghosh and Chan, 2016) to identify the positional types of the remaining transcripts. Transcripts labeled with positional codes "i" (intronic), "u" (intergenic), "x" (exonic overlap opposite strand), or "o" (exonic overlap same strand) are retained for downstream analysis.

(2). Select transcripts with length >200bp and exon number >=2.

(3). The transcripts obtained in the second step were used to predict the line coding capacity by four software (or databases), including CPC2 (Kang et al., 2017), CNCI (Sun et al., 2013), PLEK (Li et al., 2014) and Pfam (Sonnhammer et al., 1998). The transcripts in the intersection part of the above four software were filtered out to obtain the candidate IncRNA.

The selection thresholds of the four software are as follows:

1) CPC2threadhold=0, when score<0, Noncoding.

2) CNCI threadhold=0, when score<0, Noncoding.

3) PLEK threadhold=0, when score<0, Noncoding.

4) Pfam Noncoding when comparison is not possible.

(4). If the experimental species has a well-established lncRNA database, we perform BLAST alignment of the candidate lncRNAs against the known database. Candidate lncRNAs with significant matches are filtered out, and the remaining transcripts are identified as novel lncRNAs.

**1.3 Supplementary Note S3: *Differential Expression Analysis Steps Using DESeq.***

1.The estimateSizeFactors function is applied to normalize counts, generating base mean values.

2.Use the nbinomTest function to perform the test of significance and calculate the pValue and foldChange values for the comparison of differences.

3. pValue corrected by multiple hypothesis testing using the false discovery rate (FDR) method.

4.The default difference screening condition was p Value<0.05 and fold Change >2 (or <0.5).

**1.4 Supplementary Note S4: *CircRNA prediction method.***

CircRNA prediction follows the CIRI (Gao et al., 2015) process.

1. Obtain sam files by comparing clean reads to the reference species genome via BWA MEM.

2. Detect balanced jumnction reads based on xS/HyM (upstream) or xMyS/H (downstream) PCC signals.

3. Filter junction reads based on PEM and GT-AG signals.

4. Detect umbalanced junction reads using DM algorithm.

# 2 Supplementary Figure

## 2.1 Supplementary Figure S1

**Supplementary Figure S1.** Fingerprint of the Zhuyu pill and its formulae. (A-C) Three ZYP technical repetitions in HPLC analyses. a) berberine; b) coptisine; c) evodiamine; d) rutaecarpin; e) other unknown materials.

**References**

GAO, Y., WANG, J. & ZHAO, F. 2015. CIRI: an efficient and unbiased algorithm for de novo circular RNA identification. *Genome Biol,* 16**,** 4.

GHOSH, S. & CHAN, C. K. 2016. Analysis of RNA-Seq Data Using TopHat and Cufflinks. *Methods Mol Biol,* 1374**,** 339-61.

KANG, Y. J., YANG, D. C., KONG, L., HOU, M., MENG, Y. Q., WEI, L. & GAO, G. 2017. CPC2: a fast and accurate coding potential calculator based on sequence intrinsic features. *Nucleic Acids Res,* 45**,** W12-w16.

LI, A., ZHANG, J. & ZHOU, Z. 2014. PLEK: a tool for predicting long non-coding RNAs and messenger RNAs based on an improved k-mer scheme. *BMC Bioinformatics,* 15**,** 311.

SONNHAMMER, E. L., EDDY, S. R., BIRNEY, E., BATEMAN, A. & DURBIN, R. 1998. Pfam: multiple sequence alignments and HMM-profiles of protein domains. *Nucleic Acids Res,* 26**,** 320-2.

SUN, L., LUO, H., BU, D., ZHAO, G., YU, K., ZHANG, C., LIU, Y., CHEN, R. & ZHAO, Y. 2013. Utilizing sequence intrinsic composition to classify protein-coding and long non-coding transcripts. *Nucleic Acids Res,* 41**,** e166.
